# Biosynthetic engineering of the antifungal, anti-MRSA auroramycin

**DOI:** 10.1186/s12934-019-1274-y

**Published:** 2020-01-06

**Authors:** Wan Lin Yeo, Elena Heng, Lee Ling Tan, Yi Wee Lim, Kuan Chieh Ching, De-Juin Tsai, Yi Wun Jhang, Tsai-Ling Lauderdale, Kak-Shan Shia, Huimin Zhao, Ee Lui Ang, Mingzi M. Zhang, Yee Hwee Lim, Fong T. Wong

**Affiliations:** 1 Metabolic Engineering, Functional Molecules & Polymers, Institute of Chemical and Engineering Sciences, A*STAR, Biopolis, Singapore; 2 Molecular Engineering Laboratory, Institute of Bioengineering and Nanotechnology, A*STAR, Biopolis, Singapore; 3 Integrated Bio & Organic Chemistry, Functional Molecules & Polymers, Institute of Chemical and Engineering Sciences, A*STAR, Biopolis, Singapore; 4 National Institute of Infectious Diseases and Vaccinology, National Health Research Institutes (NHRI), Zhunan, Miaoli, Taiwan; 5 Institute of Biotechnology and Pharmaceutical Research, National Health Research Institutes (NHRI), Zhunan, Miaoli, Taiwan; 6 Departments of Chemical and Biomolecular Engineering, Chemistry, Biochemistry, University of Illinois at Urbana-Champaign, Urbana, Illinois USA; 7 Present Address: Institute of Molecular and Genomic Medicine, National Health Research Institutes, Zhunan, Miaoli, Taiwan

**Keywords:** Combinatorial biosynthesis, *Streptomyces*, Polyene macrolactam

## Abstract

Using an established CRISPR-Cas mediated genome editing technique for streptomycetes, we explored the combinatorial biosynthesis potential of the auroramycin biosynthetic gene cluster in *Streptomyces roseosporous.* Auroramycin is a potent anti-MRSA polyene macrolactam. In addition, auroramycin has antifungal activities, which is unique among structurally similar polyene macrolactams, such as incednine and silvalactam. In this work, we employed different engineering strategies to target glycosylation and acylation biosynthetic machineries within its recently elucidated biosynthetic pathway. Auroramycin analogs with variations in C-, N- methylation, hydroxylation and extender units incorporation were produced and characterized. By comparing the bioactivity profiles of five of these analogs, we determined that unique disaccharide motif of auroramycin is essential for its antimicrobial bioactivity. We further demonstrated that C-methylation of the 3, 5-*epi*-lemonose unit, which is unique among structurally similar polyene macrolactams, is key to its antifungal activity.

## Introduction

Natural products (NPs) are an important source for pharmacological applications with a significant proportion of current drugs being natural product or natural product-derived [1]. Advances in genome editing and synthetic biology tools, together with natural product biosynthesis knowledge accumulated over the decades, allow us to better predict, design and build pathways towards the synthesis of natural products [2]. Earlier, we established a rapid and efficient CRISPR-Cas9 strategy for biosynthetic gene cluster (BGC) editing and activation in streptomycetes [3, 4], which opens up opportunities for combinatorial biosynthesis in native streptomycete hosts [5]. Compared to chemical syntheses, combinatorial engineering of native biosynthetic pathways allows us to design and biosynthesize structurally complex chemical analogs without traversing difficult multi-step and possibly low-yielding chemical reactions, thus facilitating elucidation of structure–activity relationships towards an optimized drug lead.

**Fig. 1:**
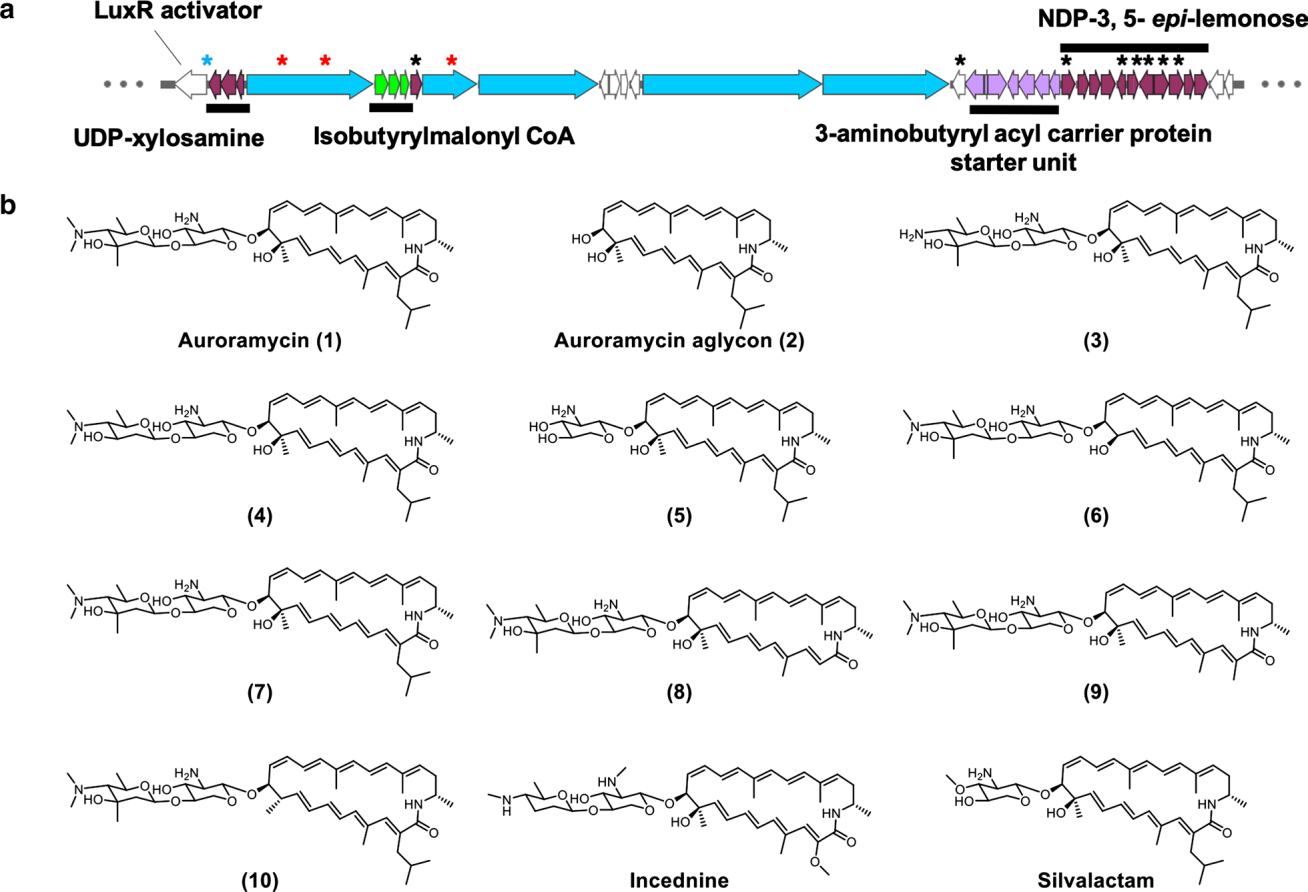
**a** Biosynthetic gene cluster and **b** structures of auroramycin, its analogs produced in this study, along with structurally related polyene macrolactams, incednine and silvalactam. The different gene manipulations on the BGC are annotated by *. Blue * for promoter insertion, red * for site mutation and black * for in-frame gene deletion. Operons for the various biosynthetic pathways are also annotated and color-coded; red is for glycosylation and sugar biosynthesis pathways, green is for extender unit biosynthesis, blue is for polyketide synthases and purple is for starter unit biosynthesis. The gene table corresponding to the gene cluster is also available as Additional file 1: Table S1 and detailed biosynthetic schemes can be found in [6]

In this study, we describe our efforts to engineer the BGC of antimicrobial auroramycin (Fig. 1a, [6, 7]) and characterize its structure activity relationship (SAR). Auroramycin (**1**) is a polyene macrolactam that is doubly glycosylated. Sugars are attached in the order of xylosamine and 3, 5-*epi*-lemonose to the polyketide core. Compared to structurally similar natural products (Fig. 1b), such as the doubly glycosylated incednine [8] and monoglycosylated silvalactam [9], auroramycin is the only polyene macrolactam with reported antifungal activity to date. One of the main structural differences between auroramycin, incednine and silvalactam is their glycosylation pattern (Fig. 1b). Glycosylation can significantly increase the diversity and complexity of natural products and has often been shown to directly and significantly impact the their bioactivity and pharmacological properties [10, 11]. As such, glycodiversification is an attractive strategy to diversify and optimize bioactivity of NPs. Auroramycin also has a unique isobutyrylmalonyl (ibm) moiety incorporated into its polyene core. This ibm extender unit is relatively rare among NPs [12]. As a mixture of malonyl and methylmalonyl moieties are also incorporated into the polyketide, acyltransferase engineering would also allow us to quickly access new structurally diverse analogs.

Here, we describe pathway engineering for glycosylation and extender unit incorporation in native *S. roseosporous*. To examine importance of polyketide backbone modifications and its unique disaccharide moiety towards anti-MRSA and antifungal activities, five new auroramycin analogs were produced and characterized (Fig. 1).

## Results

### Engineering sugar biosynthesis

**Fig. 2:**
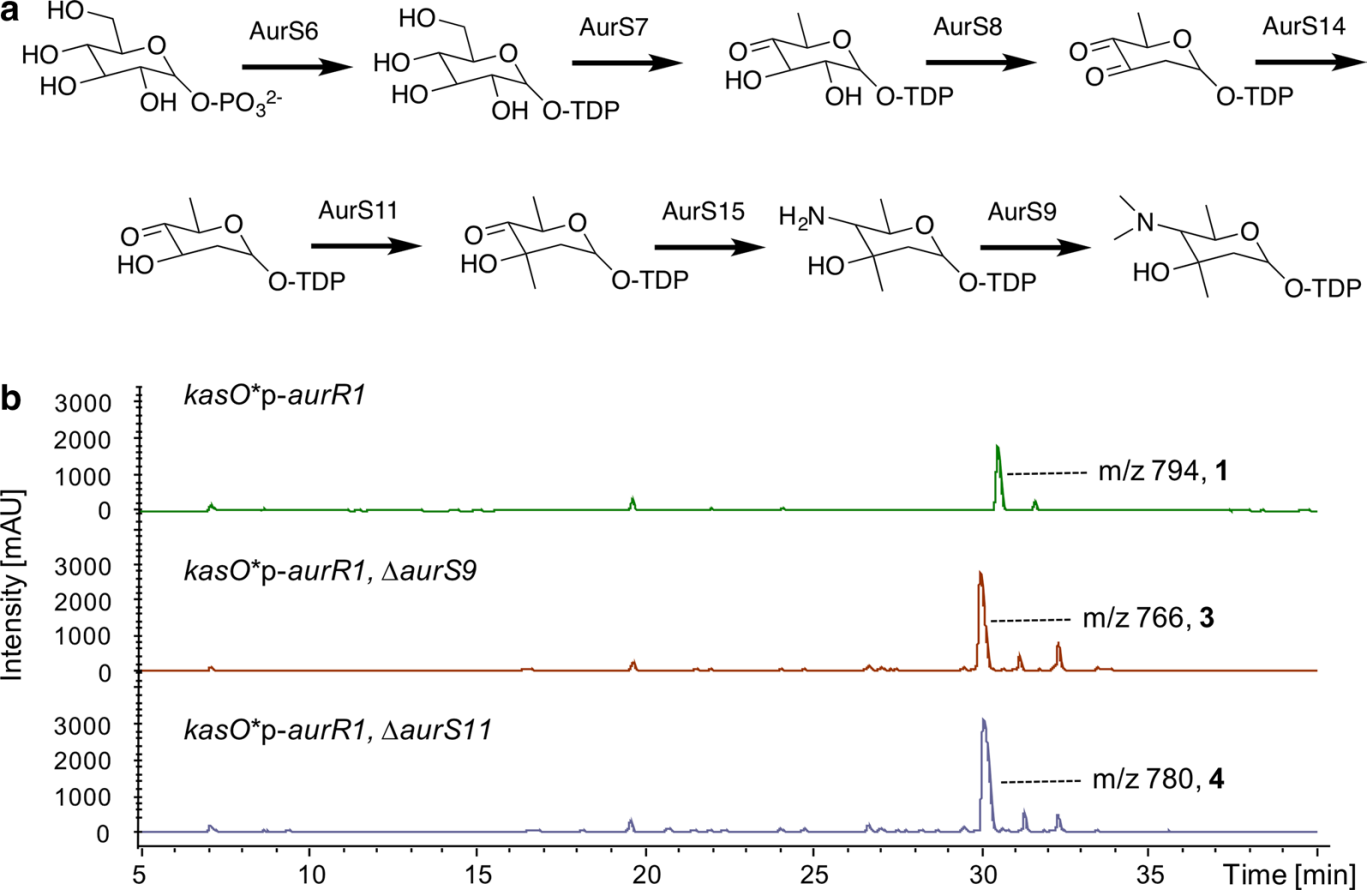
Engineering 3, 5-*epi*-lemonose biosynthesis. **a** Putative sugar biosynthesis pathway. **b** Liquid chromatography mass spectrometry (LCMS) spectra of extracts from engineered *S. roseosporus* strains with no modification to native BGC, (top) deletion of *aurS9* and (middle) deletion of *aurS11* (bottom) in the auroramycin BGC

We previously proposed the sugar biosynthesis pathways for auroramycin (Fig. 2a, [6]). Based on this, deletion of the *N*,*N*-dimethyltransferase AurS9 and C-methyltransferase AurS11 should remove N-methylation and C-methylation of 3, 5-*epi*-lemonose to yield compounds **3** and **4** respectively. As expected, **3** and **4** were main products of the respective engineered strains (Figs. 1b, 2b). Production yields of **3** and **4** were 40–80 mg/L, which are comparable to that of auroramycin. The latter observation suggested that the glycosyltransferase for 3, 5-*epi*-lemonose was able to transfer different non-methylated sugars onto the auroramycin scaffold just as efficiently as its cognate sugar substrate.

### Engineering glycosylation

**Fig. 3:**
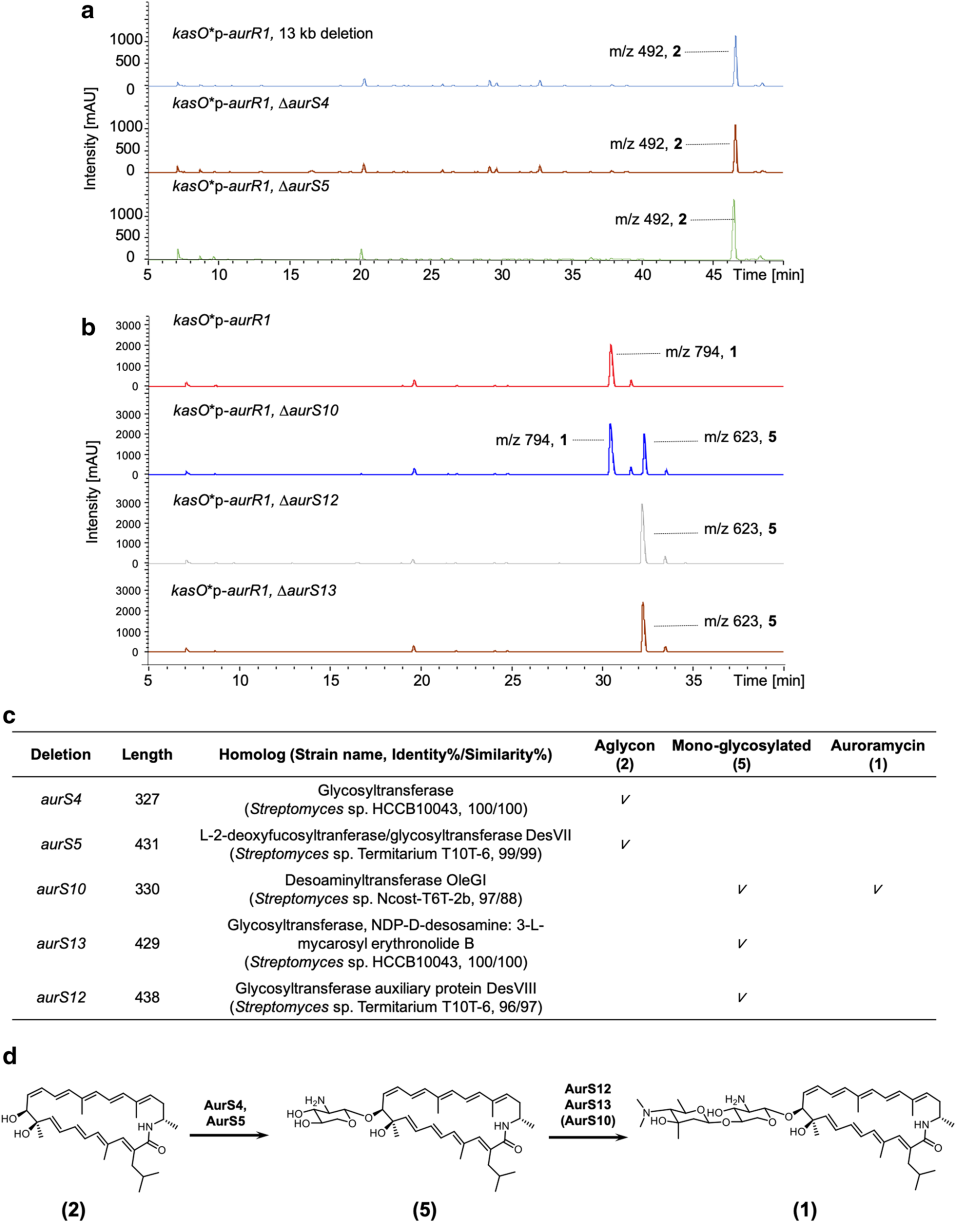
Engineering glycosylation. **a** LCMS spectra of engineered *S. roseosporus* strains with 13 kb and *aurS4* deletions within the BGC. **b** LCMS spectra of engineered *S. roseosporus* strains with no deletion, *aurS10, aurS12* and *aurS13* deletions within the BGC. **c** Table depicting the genes that were deleted, their annotated gene products and resulting metabolite produced by *S. roseosporus* strains carrying the deletion. Length refers to the number of amino acid residues of the indicated gene product. **d** Putative glycosylation scheme of auroramycin. AurS10 is not essential for auroramycin glycosylation but increases the efficiency of the second glycosylation reaction

Two glycosylation events take place during auroramycin biosynthesis. However within the BGC, there are four annotated glycosyltransferases (AurS4, S5, S10, S13) in addition to a P450 (AurS12) as its potential auxiliary partner, DesVIII [13, 14]. The four glycosyltransferases have high similarity to DesVII/EryCIII glycosyltransferases which require activation and stabilization by an DesVIII homolog auxiliary partner. A DesVII/DesVIII complex is required for activity. Of these four glycosyltransferases, two were truncated and hypothesized to be inactive; N-terminal helices, hypothesized for DesVIII interactions, and potential sites for substrates and sugar interactions were missing or modified (Additional file 1: Figure S9, [13, 14]). Earlier, we obtained aglycon **2** by deleting a 13 kb region in the BGC, which includes the *aurS5*, *S10, S12, S13* genes [6]. To assign the roles of individual glycosyltransferases and putative auxiliary partner, as well as produce the mono-glycosylated analog **5** for SAR studies, we systematically deleted each of the genes and characterized the products of the engineered *S. roseosporus* strains (Fig. 3).

Individual deletions of the 5 genes involved in auroramycin glycosylation revealed that different sets and interactions of glycosyltransferases are required for each of the two glycosylation events (Fig. 3a–c). The first glycosylation event required the gene products of *aurS4* and *aurS5* as deletion of either gene yielded aglycon **2** (Fig. 3a). The second glycosylation event likely involved a more traditional DesVII/VIII complex encoded by *aurS12* and *aurS13*. Purification and characterization of co-expressed AurS12 and AurS13 revealed that AurS13 and AurS12 have similar oligomerization profile as the DesVII/DesVIII complex [13, 14], suggesting that the two proteins form a functional glycosylation complex similar to the latter (Additional file 1: Figure S10). Deletion of either *aurS12* and *aurS13* yielded the mono-glycosylated analog **5** (Fig. 3b). As aglycon **2** was not observed with *aurS12* deletion, this suggested that the first glycosylation step did not require an auxiliary protein partner for activity. AurS10 was also not essential for auroramycin glycosylation but most likely enhanced the second glycosylation event since deletion of *aurS10* resulted in the production of a mixture of analog **5** and auroramycin (Fig. 3b).

Based on these observations, we proposed the following scheme for glycosylation in the auroramycin biosynthetic pathway: xylosamine is first glycosylated by AurS4 and AurS5, after which, 3, 5-*epi*-lemonose is added by AurS12 and AurS13 with AurS10 needed for increased efficiency of the second glycosylation step (Fig. 3c, d). Notably, contrary to in silico predictions that the truncated gene products of *aurS4* and *aurS10* are non-functional (Additional file 1: Figure S9, [15]), deletion of AurS4 and AurS10 has a profound effect on auroramycin’s glycosylation, suggesting a functional role for these truncated gene products.

### Engineering extender unit

**Fig. 4:**
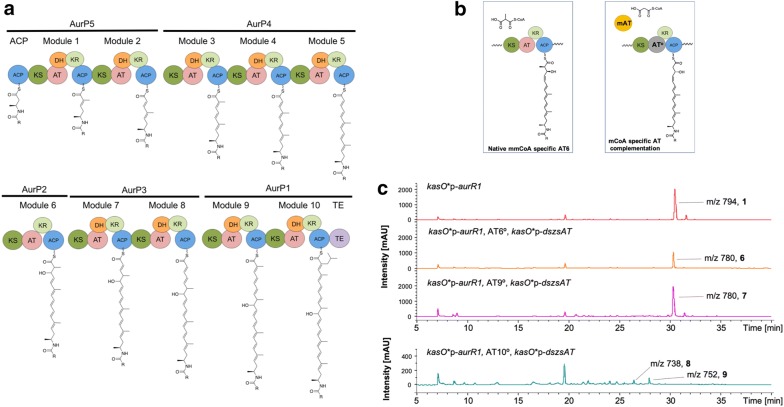
Extender unit engineering of the auroramycin PKS. **a** Polyketide synthase assembly. **b** AT complementation schematic (left: native AT in modular PKS, right, complementation of inactivated AT by trans-acting AT). **c** LCMS spectra of the auroramycin-producing strain compared to AT6°, AT9° and AT10° strains complemented by *dszsAT*. *KS* ketosynthase, *AT* acyltransferase, *KR* ketoreductase, *ACP* acyl carrier protein, *DH* dehydratase, *ER* enoyl reductase, *TE* thioesterase, *mAT* malonyl CoA-specific acyltransferase

To engineer the extender units that constitute the macrocycle core (Figs. 1, 4), we first examined a strategy consisting of complementation of an inactivated *cis*-acyltransferase with a *trans*-acting acyltransferase of a different specificity (Fig. 4a, b, [16]). Previous AT complementation examples include complementation of a single module (DEBS Mod6) for the production of 2-desmethyl-6-dEB by malonyltransferase [17] and by a *trans*-acting AT from bryostatin PKS [18]. These studies demonstrated targeted incorporation of malonyl CoA (mCoA) into the polyketide carbon backbone. To engineer the macrocycle core of auroramycin, we chose a highly active malonyl CoA-specific *trans*-acting acyltransferase from the disorazole PKS (Dszs AT, [19]) to functionally rescue inactivated methylmalonyl CoA (mmCoA)-specific and ibmCoA-specific acyltransferases in the auroramycin PKS (Fig. 4b).

Dszs AT complementation of inactive AT6 and AT9 (AT6°, AT9°) yielded the expected products **6** and **7** respectively (Figs. 4c, 1b). However, yields differed significantly with **6** produced at 50% yield compared to **7** (Fig. 4c). The latter observation was most likely due to substrate specificities of the downstream modules that limited their abilities to process the non-native intermediates [20–22]; the intermediate of AT6° required processing along four modules compared to the AT9° intermediate, which had to be accepted by a single module before cyclization.

With Dszs AT complementation of inactive AT10 (AT10°), instead of obtaining only the malonyl moiety incorporated compound (**8**, Fig. 4c), we also found the mmCoA analog **9**. Analogs **8** and **9** were produced at an approximately 1:2 ratio with yields less than 5% of auroramycin. Substrate preference of Dszs AT for mCoA to mmCoA was observed previously to be approximately more than 46,000-fold [23]. Thus the significant decrease in product yields, along with the observed mmCoA-incorporated product, suggested that C-2 methylation is highly favoured by downstream gatekeeper domains, in particular the thioesterase domain [24]. Due to the low yields of analogs **8** and **9**, their bioactivity was not characterized.

### Post PKS hydroxylation

**Fig. 5:**
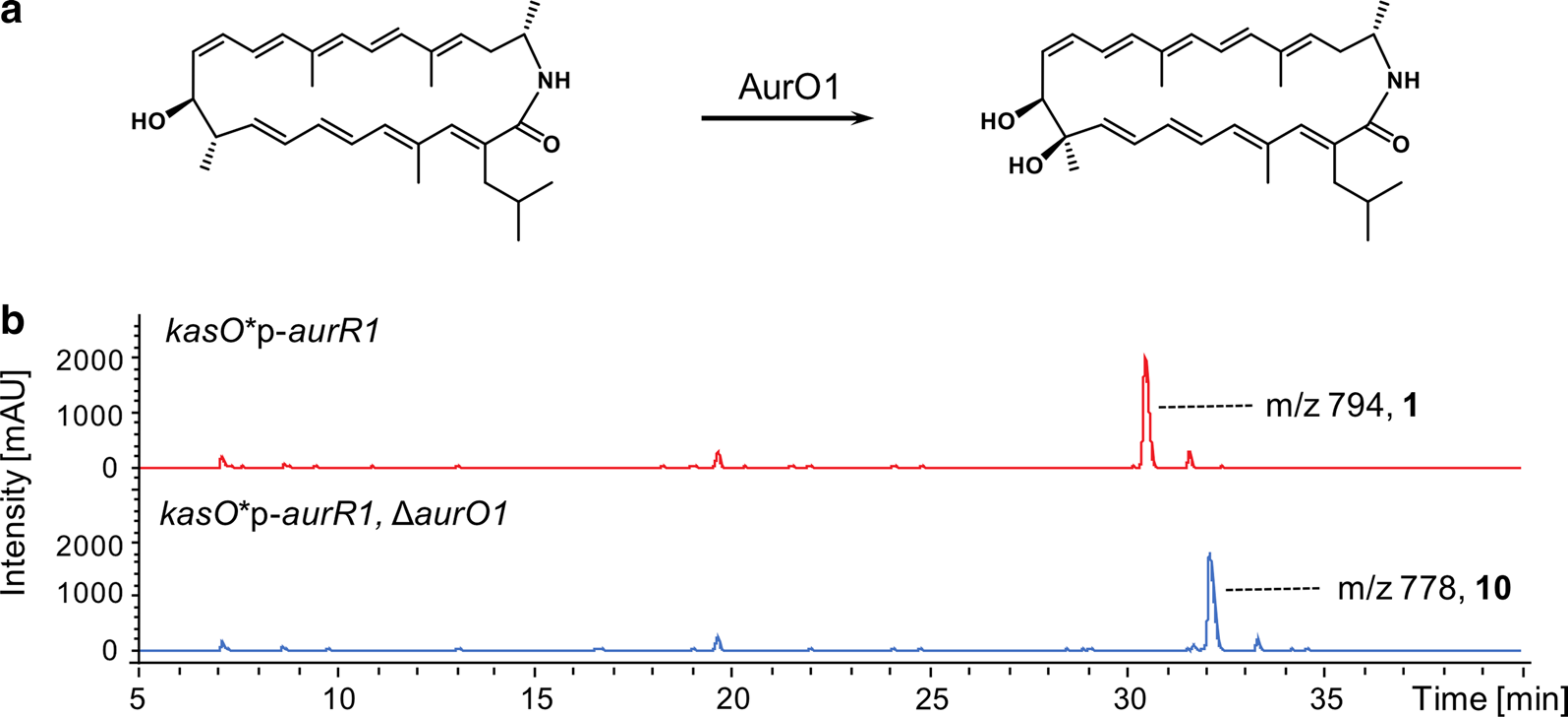
Post PKS hydroxylation. **a** Proposed hydroxylation reaction and **b** respective LCMS of the engineered *S. roseosporus* in comparison to the auroramycin producing strain

Among structurally similar natural products such as incednine, silvalactam and auroramycin (Fig. 1b), post-PKS hydroxylation takes place at a methylated site on the polyene core carbon skeleton. Conservation of this functional group suggests that either hydroxylation or/and methylation at this site might be important for bioactivity. To investigate the role of the additional hydroxyl group at C-10, we examined the production of dehydroxylated analog **10**. In the *aurO1* deletion mutant, only production of the dehydroxylated analog **10** was observed (Fig. 5).

### Antimicrobial activities of auroramycin analogs

The antifungal activities of five analogs generated in this study were compared to auroramycin against six fungal and yeast species (Table 1). Analogs **3** and **4**, which had perturbations in sugar biosynthesis, gave drastically different results in their bioactivities. The de-*N*-methylated analog **3** retains most of its bioactivity compared to auroramycin. Notably, new bioactivity (against *Kodameae ohmeri*) was observed with analog **3**, suggesting that removing the *N*-methylation on the 3, 5-*epi*-lemonose in auroramycin may improve its potential as a broad spectrum antifungal. In contrast, analog **4** is inactive against the tested fungal and yeast strains, highlighting the importance of the C-methylation on the 3, 5-*epi*-lemonose in auroramycin. As predicted, the mono-glycosylated analog **5**, which is most structurally similar to silvalactam lost all antifungal activity. Our results corroborated previous findings that silvalactam is inactive against yeast *Saccharomyces cerevisiae* [9], and further highlighted the importance of having the additional outer sugar for antifungal bioactivity. Interestingly, analogs **6** and **10**, which contained modifications at C-10 of the macrolactam core, had differing outcomes in their bioactivities. Removal of the hydroxyl group at C-10 (analog **10**) largely retained its bioactivity whereas removal of the methyl group at C-10 (analog **6**) led to complete loss of bioactivity against the tested species.

**Table 1:** Antifungal activity of auroramycin and its analogs

Compound	Fungi (MIC, µg/mL)				Yeast (MIC, µg/mL)	
	*Candida glabrata*	*Cryptococcus neoformans*	*Candida tropicalis*	*Candida albicans* —*azoles resistant*	*Saccharomyces cerevisiae*	*Kodameae ohmeri*
	ATCC 36583	ATCC 24067	ATCC 200956	ATCC 200918	ATCC 9763	CDC AR-BANK#0396
**1**	2	2	1	2	4	> 128
**3**^a^	2	1	1–2	2	2–4	2–4
**4**	> 128	> 128	64	> 128	> 128	> 128
**5**	> 128	64	> 128	> 128	> 128	>128
**6**	> 128	32	> 128	> 128	> 128	> 128
**10**	2	1	2	2	2	> 128

Minimum inhibitory concentration (MIC) refers to lowest concentration of an agent that completely inhibits visible growth in vitro of the microorganism after 2 days at 28 °C for *Saccharomyces cerevisiae* and *Kodameae ohmeri*, and at 36 °C for *Candida glabrata*, *Cryptococcus neoformans*, *Candida tropicalis* and *Candida albicans*

^a^The compound **3** tested in this MIC was isolated at a 7:1 ratio with analog **5**. As analog **5** was inactive in the assays, the bioactivity observed for compound **3** is attributed to analog **3**

We also tested antibacterial activities of the five auroramycin analogs against three Gram-positive bacteria strains, namely methicillin-resistant *Staphylococcus aureus* (MRSA), vancomycin-intermediate methicillin-resistant *Staphylococcus aureus* (VI-MRSA) and vancomycin-resistant *Enterococcus faecalis* (VRE) (Table 2). While there were some notable trends for the antibacterial activities of the analogs, there were distinct differences between the antifungal and antibacterial activities exhibited by the auroramycin analogs. While the analogs generally exhibited either clear retention or complete loss of antifungal bioactivity, alterations to the antibacterial activities for the analogs were more modest. Analog **3** retained its antibacterial activity whereas analog **4** showed some loss of activity, especially against VI-MRSA. This reflected a similar but less drastic effect of the sugar biosynthesis perturbations on antibacterial activity compared to antifungal activity. Even without its outer 3, 5-*epi*-lemonose sugar, analog **5** retained partial antibacterial activity. While modifications at C-10 of the macrolactam core gave differing outcomes in analogs **6** and **10** antifungal bioactivities, antibacterial bioactivities for analogs **6** and **10** are generally significantly reduced. Like auroramycin, the analogs are inactive against Gram-negative bacteria (MIC > 128 μg/mL).

**Table 2:** Antibacterial activity of auroramycin and its analogs

Compound	MIC, μg/mL		
	*Staphylococcus aureus* (MRSA)	*Staphylococcus aureus* (VI-MRSA)	*Enterococcus faecalis* (VRE)
	Clinical N216	Clinical Z172	ATCC 51299
**1**	4	8	2–4
**3**^a^	4	4–8	4
**4**	8–16	64	8
**5**	4–8	16	8
**6**	> 128	> 128	> 128
**10**	32	> 128	16

Minimum inhibitory concentration (MIC) refers to lowest concentration of an agent that completely inhibits visible in vitro growth of the indicated microorganism after 20 h incubation at 35 °C

^a^Compound **3** tested in this MIC was isolated at a 7:1 ratio with analog **5**

## Discussion

Among structurally similar polyene macrolactams, auroramycin is the only one with reported antifungal activity to date. Furthermore, chemogenomic analyses in yeast also determined that auroramycin’s ergosterol-independent mode of action is distinct from common antifungal polyenes, such as amphotericin, nystatin, which are known to bind to ergosterols [7]. To identify the functional groups responsible for endowing auroramycin with antifungal and anti-MRSA activities, we sought to engineer auroramycin analogs and characterize the effect of specific chemical structural changes on their bioactivity profiles. Here we targeted sites unique to auroramycin (C, N-methylation on sugars) as well as sites that are common among auroramycin, silvalactam and incednine (unique extender units and hydroxylation). Through rational engineering and CRISPR-Cas mediated genome editing, we could rapidly design, build and test different *S. roseopsorus* mutants. In our study, production yields close to that of auroramycin were achieved for most of the analogs. This could be contributed to minimal disruption to the three-dimensional structure of the polyketide assembly line and sufficiently promiscuous *cis*-acting glycosylation and sugar biosynthesis enzymes. However, there are still engineering bottlenecks, as in the case of swapping the extender unit on C-2 on auroramycin, where minimal product was observed. To access analogs restricted by downstream gatekeeper domains, strategies such as directed evolution or rational design of downstream enzymes to increase substrate promiscuity will have to be examined [25, 26]. However, these strategies are not trivial and should be undertaken for natural products of high interest. Further diversity for targeted functional groups can be achieved by altering enzyme specificities and swapping enzyme domains [16, 27].

**Fig. 6:**
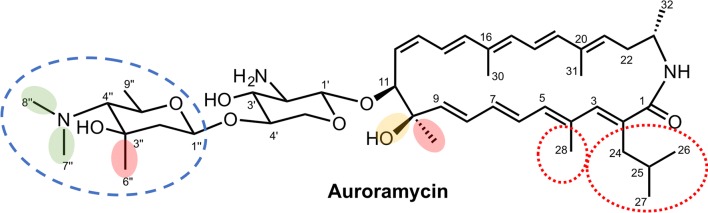
Structural activity map of auroramycin. Red shaded: removal resulted in drastic loss of both antifungal and antibacterial bioactivities; green shaded: removal resulted in retention of activity or slightly better antifungal activity, including new bioactivity against *K. ohmeri*; yellow shaded: minimal changes in antifungal activity but loss in potency in antibacterial activity; dashed blue circle: essential for antifungal activity; enhanced antibacterial potency; dotted red circle: due to the low production yields and purities, biological activity of these analogs were not functionally characterized

By comparing the bioactivity profiles of the five analogs that were achieved in good yields and purities, we were able to evaluate the functional importance of different chemical moieties on auroramycin (Fig. 6). From our studies, the unique disaccharide moiety was essential for antifungal and anti-MRSA bioactivity, however anti-MRSA activity can be restored with mono-glycosylation. Interestingly, our results demonstrated unexpected importance of the C-methylation on the outer sugar on bioactivity, especially for antifungal activity. The C-methylation on the outer sugar is unique to auroramycin and could explain auroramycin being the sole antifungal agent among similar polyene macrolactams. Perturbation of the macrolactam core of auroramycin led to mixed outcomes for antifungal activity but mostly resulted in loss of antibacterial activity. Overall, the structural features examined in our study defined auroramycin’s antifungal bioactivity more distinctly than its antibacterial activity. Additional modifications to the sugars and the degree of saturation of the polyene macrolactam core may be explored to improve the antifungal activity of auroramycin.

Due to highly efficient, precise and consistent CRISPR-Cas mediated genome editing and advanced DNA assembly methods [28, 29], we were able to accelerate the generation of 12 strains (with at least two genomic edits each) for analog production and screening in this study. Multiplex editing is expected to further accelerate strain construction [3]. Multiplex inactivation of key biosynthetic genes within a target BGC in streptomycetes can also be achieved with base editing using Cas9-deaminase fusions [30]. Last but not least, a major consideration of any genome editing strategy is that it requires the introduction of recombinant DNA, which may be challenging depending on the target actinomycete strain.

## Conclusion

In this work, we used CRISPR-Cas mediated genome editing to accelerate the rational engineering of the antimicrobial auroramycin biosynthetic gene cluster. From a single design–build–test cycle, we were able to make specific changes to the glycosylation pattern and polyene macrolactam core of auroramycin to generate analogs. Five of these analogs were biosynthesized in good yields and their bioactivities were further characterized. By comparing the bioactivities of these analogs, we determined that the unique disaccharide moiety, in particular C-methylation of the outer sugar unit, was important for the antifungal activity of auroramycin.

## Materials and methods

### Growth and conjugation conditions

Unless otherwise indicated, all reagents are obtained from Sigma, St. Louis, USA. Conjugation experiments involving WM3780 *E. coli* strains were performed on R2 agar without sucrose. Unless otherwise indicated, strains are propagated in MGY medium at 30 °C. Spore preparations and conjugation protocols were similar as described before [3]. A typical spore prep contains ~ 10^6^–10^7^ spores/mL as determined by serial dilution plating. List of strains and plasmids used in this study is available in Additional file 1: Table S2.

### Construction of genome editing plasmids

All DNA manipulations were carried out in OmniMAX™ (Thermo Fisher, Massachusetts, USA). Primers used in this study are listed in Additional file 2: Table S3. Restriction enzymes were obtained from New England Biolabs. Protospacers were first inserted via BbsI-mediated Golden Gate Assembly before introduction of the respective homology flanks via Gibson assembly, as previously described [3]. Detailed description of *aur*S5 deletion strain can also be found in [4].

### Engineering of strains

Precise deletions of individual target genes without affecting intergenic regions were made using a CRISPR-Cas mediated editing strategy ([3], Additional file 1: Figure S1–S7). To delete *aurS5*, we had to use a different Cas protein [4]. As described elsewhere [6], in order to activate the entire BGC for production of the corresponding analogs, *luxR* in these edited strains was also placed under a strong constitutive kasO* promoter [31].

AT domains in modules 6, 9 and 10 were independently inactivated with a single active site serine to alanine mutation (Additional file 1: Figure S8). A gene cassette of Dszs AT, placed under kasO*p, was integrated into the *attB* site of the genome [32]. The *luxR* gene within the BGC in these strains was also under kasO*p to activate its production.

### Validation of promoter knock-in and genome editing

Genomic DNA from wild type and exconjugants from the indicated strains were isolated from liquid cultures using the Blood and Tissue DNeasy kit (Qiagen, Hilden, Germany) after pretreating the cells with 20 mg/mL lysozyme for 0.5–1 h at 30 °C. PCR was performed using control primers beyond the homology regions with KODXtreme *Taq* polymerase (Millipore, Massachusetts, USA). Where indicated, PCR products were subjected to digest with specific restriction enzymes to differentiate between PCR products of wild type genomic sequences and successful genome editing by knock-ins. Positive samples were purified using ExoSAP-IT™ (Affymetrix USB, Massachusetts, USA) and validated by Sanger sequencing.

### Fermentation

Fermentation of analogs and auroramycin was performed as described elsewhere [6] using the indicated engineered *Streptomyces roseosporus* NRRL 15998 strains.

### Isolation and characterization of analogs

#### General considerations

Optical rotation was obtained on a JASCO P1030 polarimeter using a micro-cell (light path 1 cm). IR spectrum was taken on a PerkinElmer Spectrum 100 FT-IR spectrometer. UV spectra were recorded on Shimadzu UV-2450 UV–Vis spectrophotometer. CD spectra were taken on a JASCO J-810 CD Spectropolarimeter. HRMS spectra were measured on an Agilent 6545 Quadrupole-Time-of-Flight (Q-TOF) mass spectrometer. ^1^H, ^13^C, and 2D-NMR spectra were recorded on a Bruker 400 MHz and Varian VNMRS 700 MHz spectrometers and calibrated using residual non-deuterated solvent (CD_2_Cl_2_: δ_H_ = 5.32 ppm, δ_C_ = 53.84 ppm) as an internal reference.

#### Analog 3

To the crude ethyl acetate extracts from 200 agar fermentation plates (~ 6 L), cold ethyl acetate (20 mL × 2) was added, sonicated for 1–2 min and centrifuged to separate the yellow solution and yellow solid. Cold methanol (10 mL) was then added to the yellow solid, sonicated and centrifuged to separate the yellow solution. The obtained white/pale yellow solid (238.0 mg, > 90% purity) was found to be mainly analog **3** together with monoglycosylated analog **5** in an approximate 7:1 ratio. See Additional file 1: Figure S11 for structural assignment for analog **3**; HRMS *m/z*: 794.5000 [(M + H)^+^ calcd. for C_44_H_68_N_3_O_8_, 766.5006]. See latter for assignment of analog **5**.

#### Analog 4

To the crude ethyl acetate extracts from 125 agar fermentation plates (~ 4 L), cold ethyl acetate (20 mL × 2) was added, sonicated for 1–2 min and centrifuged to separate the yellow solution and yellow solid. Cold methanol (15 mL) was then added to the yellow solid, sonicated and centrifuged to separate the yellow solution and analog **4** was obtained as a white/pale yellow solid (315.0 mg, > 90% purity). [α]_D_^25^: − 301.6° (*c* 0.25, 1:1 CH_2_Cl_2_:MeOH); ^1^H and ^13^C NMR: see Additional file 1: Table S4; IR (KBr): *ν* = 3353, 2953, 2925, 2871, 1630, 1577, 1525, 1459, 1384, 1284, 1234, 1167, 1129, 1079, 1058, 985, 960 cm^−1^; UV/VIS (1:1 CH_2_Cl_2_:MeOH): *λ*_max_ (log *ε*) = 317.5 (1.25) nm; HRMS *m/z*: 780.5161 [(M + H)^+^ calcd. for C_45_H_70_N_3_O_8_, 780.5163].

#### Analog 5

To the crude ethyl acetate extracts from 184 agar fermentation plates (~ 6 L), cold ethyl acetate (30 mL) was added, sonicated for 1–2 min and centrifuged to separate the yellow solution and yellow solid. Cold methanol (5 mL) was then added to the yellow solid, sonicated and centrifuged to separate the yellow solution and analog **5** was obtained as a white/pale yellow solid (92.1 mg, > 94% purity). [α]_D_^25^: − 233.5° (*c* 0.2, 1:1 CH_2_Cl_2_:MeOH); ^1^H and ^13^C NMR: see Additional file 1: Table S4; IR (KBr): *ν* = 3350, 2925, 2854, 1712, 1628, 1530, 1463, 1386, 1285, 1147, 1077, 1057, 985, 961 cm^−1^; UV/VIS (MeOH): *λ*_max_ (log *ε*) = 317.5 (1.74) nm; HRMS *m/z*: 623.4056 [(M + H)^+^ calcd. for C_37_H_55_N_2_O_6_, 623.4060].

#### Analog 6

To the crude ethyl acetate extracts from 200 agar fermentation plates (~ 4 L), cold diethyl ether (10 mL × 2) was added, sonicated for 1–2 min and centrifuged to separate the yellow solution. Analog **6** was obtained in an approximate 17:1 ratio together with a hydroxylated product as a brown solid (107 mg, > 92% purity). See Additional file 1: Figure S12 for structural assignment; HRMS *m/z*: 780.5156 [(M + H)^+^ calcd. for C_45_H_69_N_3_O_8_, 780.5157].

#### Analog 7

To the crude ethyl acetate extracts from 191 agar fermentation plates (~ 4 L), cold diethyl ether (10 mL × 2) was added, sonicated for 1–2 min and centrifuged to separate the yellow solution. Analog **7** was obtained in an approximate 5:1 ratio together with a hydroxylated product as a brown solid (165 mg, > 90% purity). See Additional file 1: Figure S13 for structural assignment; HRMS *m/z*: 780.5161 [(M + H)^+^ calcd. for C_45_H69N_3_O_8_, 780.5157]. Due to constraints of the sensitivity of the compounds to light and acid, further purification was not performed.

#### Analog 10

To the crude ethyl acetate extracts from 180 agar fermentation plates (~ 6 L), cold Et_2_O (10–15 mL × 2) was added, sonicated for 1–2 min and centrifuged to separate the yellow solution and brown solid. The brown solid was washed with cold 5:1 Et_2_O:acetone (2 mL × 3) and analog **10** was obtained as a brown solid (69.7 mg, > 94% purity). [α]_D_^25^: –261.9° (*c* 0.25, 1:1 CH_2_Cl_2_:MeOH); ^1^H and ^13^C NMR: see Additional file 1: Table S5; IR (KBr): *ν* = 3333, 2926, 2867, 1661, 1515, 1456, 1382, 1274, 1238, 1156, 1097, 1058, 988, 962 cm^−1^; UV/VIS (MeOH): *λ*_max_ (log *ε*) = 317.5 (1.90) nm; HRMS *m/z*: 778.5361 [(M + H)^+^ calcd. for C_46_H_72_N_3_O_7_, 778.5370].

### Antifungal assays

Measurements against indicated fungal strains were conducted at Eurofins Panlabs Taiwan (www.eurofins.com/epds), according to methodology described by the Clinical and Laboratory Standards Institute (CLSI) (M38-A, M27-A2).

### Bacterial assays

Minimum inhibition concentration (MIC) values were determined by the broth microdilution method, as recommended by the Clinical and Laboratory Standards Institute with slight modifications. Briefly, purified auroramycin analogs were dissolved in DMSO, then diluted in Mueller–Hinton broth containing 0.2% DMSO. The organisms were tested at 5 × 10^5^ CFU/mL. The MICs were read at 20 h after 35 °C incubation (Additional file 1: Figure S14).

## Supplementary information

**Additional file 1. Table S1.** Putative functions of genes in the auroramycin biosynthetic gene cluster. **Table S2.** List of strains and plasmids used in this study. **Table S4.** ^1^H and ^13^C NMR data of auroramycin analog **4** and analog **5**. **Tabe S5.** ^1^H and ^13^C NMR data of auroramycin analog **10**. **Figure S1.** Alignment of sanger sequencing of *aurS9* deletion mutant (edited) against wild type genome. **Figure S2.** Alignment of sanger sequencing of *aurS11* deletion mutant (edited) against wild type genome. **Figure S3.** Alignment of sanger sequencing of *aurS4* deletion mutant (edited) against wild type genome. **Figure S4.** Alignment of sanger sequencing of *aurS10* deletion mutant (edited) against wild type genome. **Figure S5.** Alignment of sanger sequencing of *aurS12* deletion mutant (edited) against wild type genome. **Figure S6.** Alignment of sanger sequencing of *aurS13* deletion mutant (edited) against wild type genome. **Figure S7.** Alignment of sanger sequencing of *aur01* deletion mutant (edited) against wild type genome. **Figure S8.** Alignment of sanger sequencing of AT mutant (edited) against wild type genome. **Figure S9** Alignment of glycosyltransferases. **Figure S10** Purification and characterization of His-tagged AurS13 expressed alone or His-tagged AurS13 co-expressed with AurS12 (no His-tag). **Figure S11.** Analytical data for the structural assignment of auroramycin analog **3**. **Figure S12.** Analytical data for the structural assignment of auroramycin analog **6** and minor hydroxylated product. **Figure S13.** Analytical data for the structural assignment of auroramycin analog **7** and minor hydroxylated product. **Figure S14.** Representative microdilution assay plates for compound **1**, **3**-**7**, **10** with (A) *Staphylococcus aureus* N216, (B) *S. aureus* Z172, (C) *Enterococcus faecalis* ATCC 51299, (D) *Pseudomonas aeruginosa* ATCC 700603 and (E) *E. coli* ATCC 25922.

**Additional file 2: Table S3.** Oligonucleotides used in this study.

## Data Availability

All data generated or analysed during this study are included in this published article [and its Additional file 1].

## Abbreviations

SAR: structure activity relationship
Ibm: isobutyrylmalonyl
Dszs: disorazole synthase
PKS: polyketide synthase
KS: ketosynthase
AT: acyltransferase
KR: ketoreductase
ACP: acyl carrier protein
DH: dehydratase
ER: enoyl reductase
TE: thioesterase
mAT: malonyl CoA-specific acyltransferase
MRSA: methicillin-resistant *Staphylococcus aureus*
HRMS: high resolution mass spectrometry
LCMS: liquid chromatography mass spectrometry
mCoA: malonyl Coenzyme A
mmCoA: methylmalonyl Coenzyme A
