# Supplementary material for: Biosynthetic engineering of the antifungal, anti-MRSA auroramycin
Source: Microb Cell Fact. 2020 Jan 6;19:3. doi: 10.1186/s12934-019-1274-y (PMC6943886; doi:10.1186/s12934-019-1274-y)
Supplement: Supplementary file 1 — Additional file 1. Table S1. Putative functions of genes in the auroramycin biosynthetic gene cluster. Table S2. List of strains and plasmids used in this study. Table S4. 1H and 13C NMR data of auroramycin analog 4 and analog 5. Tabe S5. 1H and 13C NMR data of auroramycin analog 10. Figure S1. Alignment of sanger sequencing of aurS9 deletion mutant (edited) against wild type genome. Figure S2. Alignment of sanger sequencing of aurS11 deletion mutant (edited) against wild type genome. Figure S3. Alignment of sanger sequencing of aurS4 deletion mutant (edited) against wild type genome. Figure S4. Alignment of sanger sequencing of aurS10 deletion mutant (edited) against wild type genome. Figure S5. Alignment of sanger sequencing of aurS12 deletion mutant (edited) against wild type genome. Figure S6. Alignment of sanger sequencing of aurS13 deletion mutant (edited) against wild type genome. Figure S7. Alignment of sanger sequencing of aur01 deletion mutant (edited) against wild type genome. Figure S8. Alignment of sanger sequencing of AT mutant (edited) against wild type genome. Figure S9 Alignment of glycosyltransferases. Figure S10 Purification and characterization of His-tagged AurS13 expressed alone or His-tagged AurS13 co-expressed with AurS12 (no His-tag). Figure S11. Analytical data for the structural assignment of auroramycin analog 3. Figure S12. Analytical data for the structural assignment of auroramycin analog 6 and minor hydroxylated product. Figure S13. Analytical data for the structural assignment of auroramycin analog 7 and minor hydroxylated product. Figure S14. Representative microdilution assay plates for compound 1, 3-7, 10 with (A) Staphylococcus aureus N216, (B) S. aureus Z172, (C) Enterococcus faecalis ATCC 51299, (D) Pseudomonas aeruginosa ATCC 700603 and (E) E. coli ATCC 25922. [file 12934_2019_1274_MOESM1_ESM.pdf]

Supplemental data

Biosynthetic engineering of the antifungal, anti-MRSA auroramycin

Wan Lin Yeo[1], Elena Heng[2], Lee Ling Tan[2], Yi Wee Lim[3], Kuan Chieh Ching[3], De-Juin Tsai[4], Yi Wun Jhang[4], Tsai-Ling Lauderdale[4], Kak-Shan Shia[4], Huimin Zhao[5], Ee Lui Ang[1], Mingzi M. Zhang[1,6], Yee Hwee Lim[3, #], Fong T. Wong[2, #]

1. Metabolic Engineering Research Laboratory, Functional Molecules & Polymers, Institute of Chemical and Engineering Sciences, A*STAR, Singapore
2. Molecular Engineering Laboratory, Institute of Bioengineering and Nanotechnology, A*STAR, Singapore
3. Integrated Bio & Organic Chemistry, Functional Molecules & Polymers, Institute of Chemical and Engineering Sciences, A*STAR, Singapore
4. National Institute of Infectious Diseases and Vaccinology (DJT & TLL) and Institute of Biotechnology and Pharmaceutical Research (KSS), National Health Research Institutes (NHRI), Taiwan, R.O.C.
5. Department of Chemical and Biomolecular engineering, Department of Chemistry, Department of Biochemistry, University of Illinois at Urbana-Champaign, United States
6. Current address: Institute of Molecular and Genomic Medicine, National Health Research Institutes, Taiwan, R.O.C.

# Correspondence can be directed to: lim_yee_hwee@ices.a-star.edu.sg, wongft@ibn.a-star.edu.sg

Supplemental Table S3. Oligonucleotides used in this study

- Available as Additional File 2

Supplemental Table S1. Putative functions of genes in the auroramycin biosynthetic gene cluster. The genes are colored based on their proposed roles in auroramycin biosynthesis. Red highlighted rows are for sugar biosynthesis, blue for polyketides, green for extender unit, purple for starter unit respectively. Rows that are not highlighted include regulators, transporters, thioesterase and P450. The order and color coding of the genes in this table also corresponds to gene arrangement in Figure 1A.

Gene	Homolog (Strain name, Identity %/Similarity%)	Predicted function in biosynthesis	Edit performed
aurR1	LuxR family transcriptional regulator [*Streptomyces* sp. Tue 6075, 97/98]	LuxR family transcriptional regulator	Strong promoter inserted
aurS1	nucleoside-diphosphate-sugar epimerase (UDP-glucose 4-epimerase) [*Streptomyces* sp. HCCB10043, 99/100]	NAD-dependent epimerase/dehydratase	N.A.
aurS2	nucleotide sugar dehydrogenase [*Streptomyces* sp. Tue 6075, 99/99]	UDP-glucose/GDP-mannose dehydrogenase	N.A.
aurS3	*N*-acetylglucosaminyl deacetylase, LmbE family [*Streptomyces* sp. Ncost-T6T-2b, 99/99]	LmbE family protein	N.A.
aurP1	hypothetical protein [*Streptomyces* sp. Tue 6075, 98/98]	PKS (Mod9-Mod10-TE)	Site mutagenesis of acyltransferase active site
aurM1	crotonyl-CoA reductase [*Streptomyces* sp. HCCB10043, 100/100]	crotonyl-CoA reductase	N.A.
aurM2	3-oxoacyl-[acyl-carrier-protein] synthase III [*Streptomyces* sp. HCCB10043, 100/100]	3-oxoacyl-[acyl-carrier-protein] synthase III	N.A.
aurM3	3-hydroxybutyryl-CoA dehydrogenase [*Streptomyces* sp. HCCB10043, 100/100]	3-hydroxybutyryl-CoA dehydrogenase	N.A.
aurS4	Glycosyltransferase [*Streptomyces* sp. HCCB10043, 100/100]	glycosyltransferase	Deletion
aurP2	Type I polyketide synthase [*Streptomyces* sp. Tue 6075, 97/97]	PKS (Mod6)	Site mutagenesis of acyltransferase active site
aurP3	beta-ketoacyl synthase [*Streptomyces* sp. Tue 6075, 97/97]	PKS (Mod7-Mod8)	N.A.
aurR2	TetR/AcrR family transcriptional regulator [*Streptomyces* sp. Tue 6075, 99/99]	TetR/AcrR family transcriptional regulator	N.A.
aurT1	ABC transporter ATP-binding protein [*Streptomyces* sp. HCCB10043, 99/100]	ABC-type multidrug transporter	N.A.
aurT2	ABC transporter, permease protein [*Streptomyces* sp. HCCB10043, 100/100]	ABC-type multidrug transporter	N.A.
aurA	thioesterase [*Streptomyces* sp. Tue 6075, 99/98]	Thioesterase II	N.A.
aurP4	type I polyketide synthase [*Streptomyces* sp. ML694-90F3, 61/69]	PKS (Mod3-Mod4-Mod5)	N.A.
aurP5	type I polyketide synthase [*Streptomyces* sp. ML694-90F3, 60/69]	PKS (ACP-Mod1-Mod2)	N.A.
aurO1	cytochrome P450 [*Streptomyces* sp. HCCB10043, 100/100]	Cytochrome P450	Deletion
aurL1	non-ribosomal peptide synthetase [*Streptomyces* sp. HCCB10043, 100/100]	AMP-dependent synthetase	N.A.
aurL2	phosphopantetheine-binding protein [*Streptomyces* sp. Tue 6075, 97/98]	Acyl carrier protein	N.A.
aurL3	AMP-dependent synthetase [*Streptomyces* sp. Tue 6075, 99/98]	AMP-dependent synthetase	N.A.
aurL4	Malonyl CoA-acyl carrier protein transacylase [*Streptomyces* sp. HCCB10043, 100/100]	Malonyl CoA-acyl carrier protein transacylase	N.A.
aurL5	aminotransferase [*Streptomyces* sp. Tue 6075, 98/98]	aminotransferase	N.A.
aurL6	lysine 2,3-aminomutase [*Streptomyces* sp. Tue 6075, 99/99]	lysine 2,3-aminomutase	N.A.
aurL7	proline-specific peptidase [*Streptomyces* sp. HCCB10043, 100/100]	proline iminopeptidase	N.A.
aurS5	L-2-deoxyfucosyltransferase/glycosyltransferase DesVII [*Streptomyces* sp. Termitarium-T10T-6, 99/99]	glycosyltransferase	Deletion
aurS6	glucose-1-phosphate thymidylyltransferase [*Streptomyces* sp. Termitarium-T10T-6, 100/100]	glucose-1-phosphate thymidylyltransferase	N.A.
aurS7	dTDP-glucose 4,6-dehydratase [*Streptomyces* sp. Ncost-T6T-2b, 98/99]	dTDP-glucose 4,6-dehydratase	N.A.
aurS8	NDP-hexose 2,3-dehydratase [*Streptomyces* sp. Tue 6075, 98/98]	NDP-hexose 2,3-dehydratase	N.A.
aurS9	dTDP-3-amino-3,4,6-trideoxy-alpha-D-glucopyranoseN,N-dimethyltransferase/N-dimethyltransferase [*Streptomyces* sp. Termitarium-T10T-6, 99/100]	*N*-methyltransferase	Deletion
aurS10	desosaminyltransferase OleGI [*Streptomyces* sp. Ncost-T6T-2b, 97/98]	glycosyltransferase	Deletion
aurS11	methyltransferase [*Streptomyces* sp. Tue 6075, 99/99]	NDP-hexose 3-C-methyltransferase	Deletion
aurS12	glycosyltransferase auxiliary protein DesVIII [*Streptomyces* sp. Termitarium-T10T-6, 96/97]	glycosyltransferase auxiliary protein	Deletion
aurS13	glycosyl transferase, NDP-D-desosamine: 3-L-mycarosyl erythronolide B [*Streptomyces* sp. HCCB10043, 100/100]	glycosyltransferase	Deletion
aurS14	NDP-hexose-3-ketoreductase [*Streptomyces* sp. Termitarium-T10T-6, 97/98]	NDP-hexose-3-ketoreductase	N.A.
aurS15	dTDP-4-amino-4,6-dideoxyglucose [*Streptomyces* sp. Ncost-T6T-2b, 99/99]	dTDP-4-amino-4,6-dideoxyglucose	N.A.

aurT3	transporter [*Streptomyces* sp. Tue 6075, 98/99]	ABC-type multidrug transporter	
aurT4	ABC transporter [*Streptomyces* sp. Tue 6075, 99/99]	ABC-type multidrug transporter	

Supplemental Table S2. List of strains and plasmids used in this study.

Strains and plasmids	Description	Source or/and reference
pCRISPomyces-2 (pCm2)	*AprR, oriT, rep*$^{pSG5(ts)}$*, ori*^{ColE1}*, sSpcas9,* sgRNA cassette	[1] https://www.addgene.org/61737/
pCm2-adapter1-*kasO*p*-adapter2	pCM2 with adapters to facilitate assembly of editing flanks upstream and downstream of *kasO*p*	[2]
pCRISPomyces-SaCas9	pCM2 with adapters to facilitate assembly of editing flanks upstream and downstream of *P8-kasO*p*, with Staphylococcus aureus Cas9 in place of SpCas9	[3] https://www.addgene.org/129553/
pSET152	Nonreplicative plasmid in Streptomyces with attP site and integrase gene of C31 phage	[4]
pSET152-*kasO*p*-Dszs AT	pSET152 (integration plasmid) with acyltransferase from disorazole polyketide synthase under *kasO*p*	This work
Escherichia coli OmniMAX™	*F– proAB+ lacIq lacZΔM15 Tn10(TetR) Δ(ccdAB) mcrA Δ(mrr-hsdRMS-mcrBC) Φ80lacZΔM15 Δ(lacZYA-argF) U169 endA1 recA1 supE44 thi-1 gyrA96 relA1 tonA panD*	Thermo Fisher
Escherichia coli WM3780	*dam-3 dcm-9 metB1 galK2 galT27 lacY1 tsx-78 supE44 thi-1 mel-1 tonA31 attHK*::pJK202Δ(*ori*R6K-*aadA*)::Frt	William Metcalf laboratory, [5]
Streptomyces roseosporus NRRL15998 *kasO*p-aurR1*	Auroramycin expression strain	[2]

1. Cobb, Ryan E., Yajie Wang, and Huimin Zhao. "High-efficiency multiplex genome editing of Streptomyces species using an engineered CRISPR/Cas system." ACS synthetic biology 4.6 (2014): 723-728.
2. Zhang, Mingzi M., et al. "CRISPR–Cas9 strategy for activation of silent Streptomyces biosynthetic gene clusters." Nature chemical biology 13.6 (2017): 607.
3. Yeo, Wan Lin, et al. "Characterization of Cas proteins for CRISPR-Cas editing in streptomycetes." Biotechnology and bioengineering (2019).
4. Sioud, Samiha, et al. "Integrative gene cloning and expression system for Streptomyces sp. US 24 and Streptomyces sp. TN 58 bioactive molecule producing strains." BioMed Research International 2009 (2009).
5. Blodgett, Joshua AV, Jun Kai Zhang, and William W. Metcalf. "Molecular cloning, sequence analysis, and heterologous expression of the phosphinothricin tripeptide biosynthetic gene cluster from Streptomyces viridochromogenes DSM 40736." Antimicrobial agents and chemotherapy 49.1 (2005): 230-240.

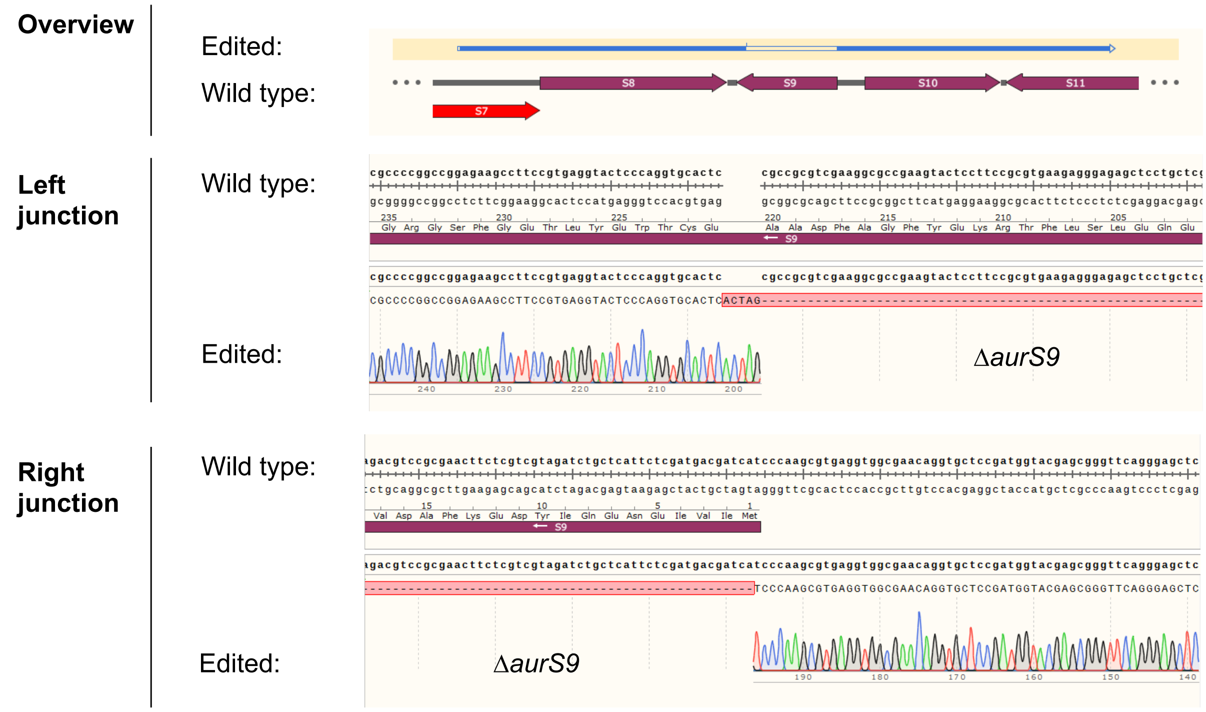

Figure S1. Alignment of sanger sequencing of *aurS9* deletion mutant (edited) against wild type genome.

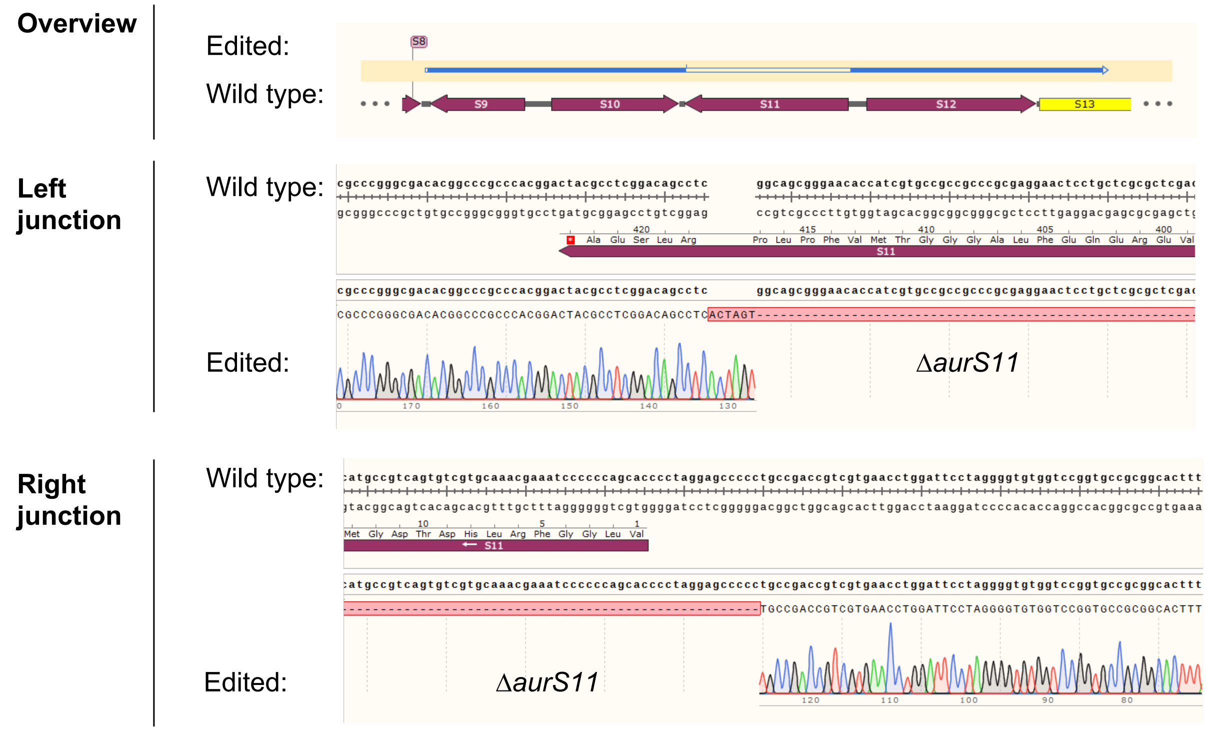

Figure S2. Alignment of sanger sequencing of *aurS11* deletion mutant (edited) against wild type genome.

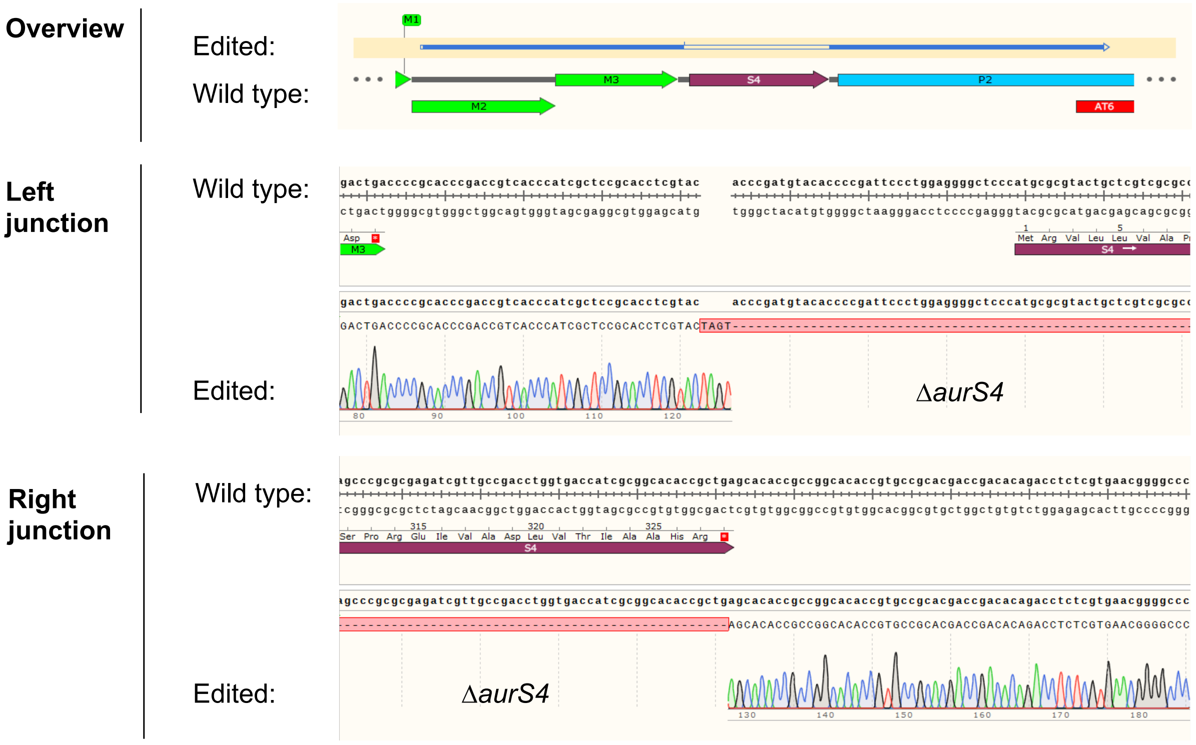

Figure S3. Alignment of sanger sequencing of *aurS4* deletion mutant (edited) against the wild type genome

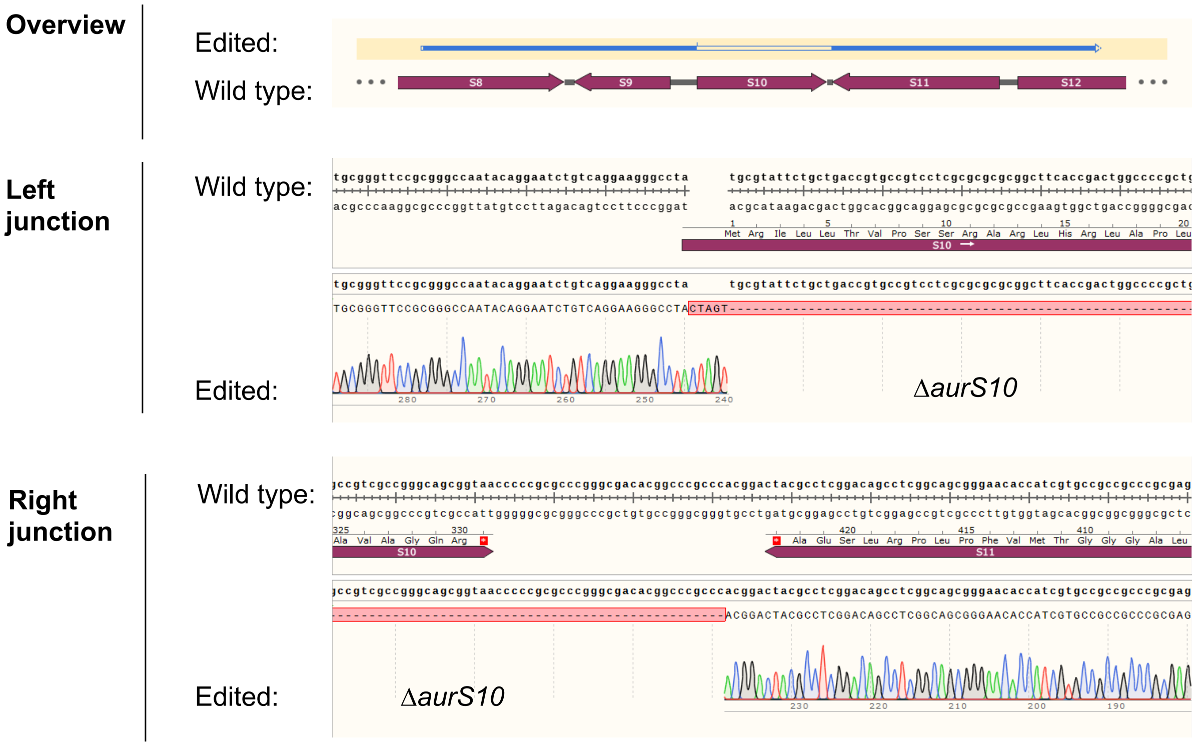

Figure S4. Alignment of sanger sequencing of *aurS10* deletion mutant (edited) against the wild type genome

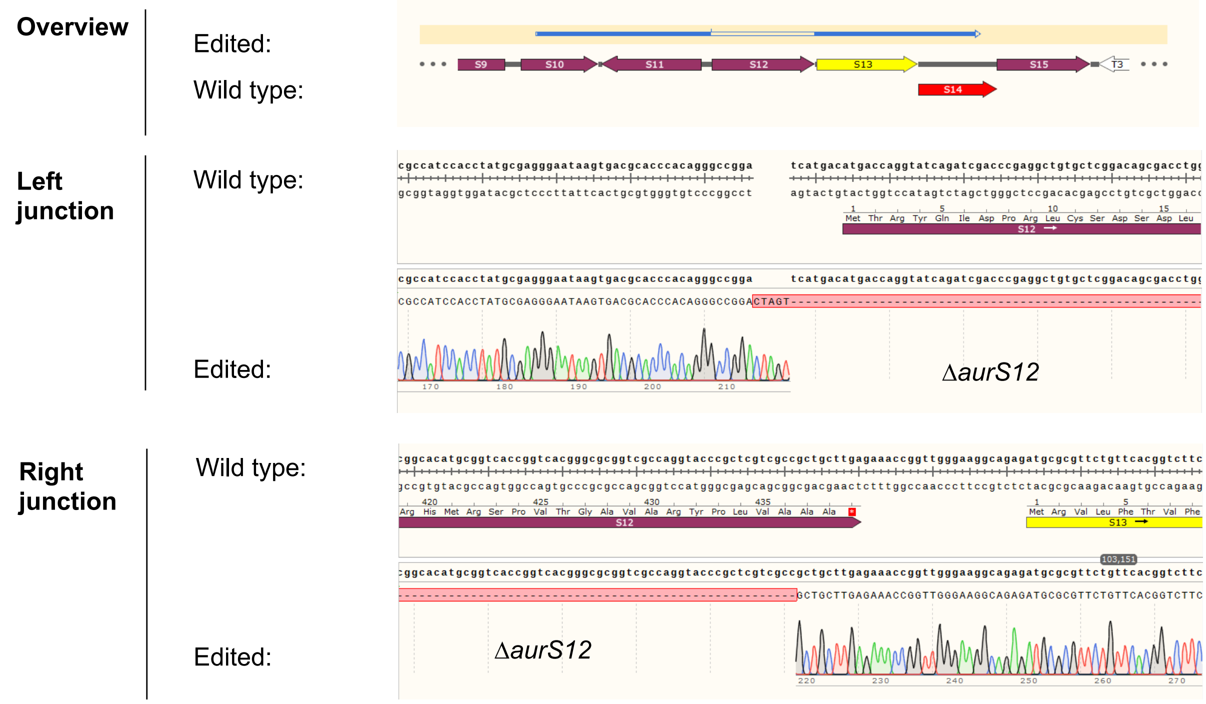

Figure S5. Alignment of sanger sequencing of *aurS12* deletion mutant (edited) against wild type genome.

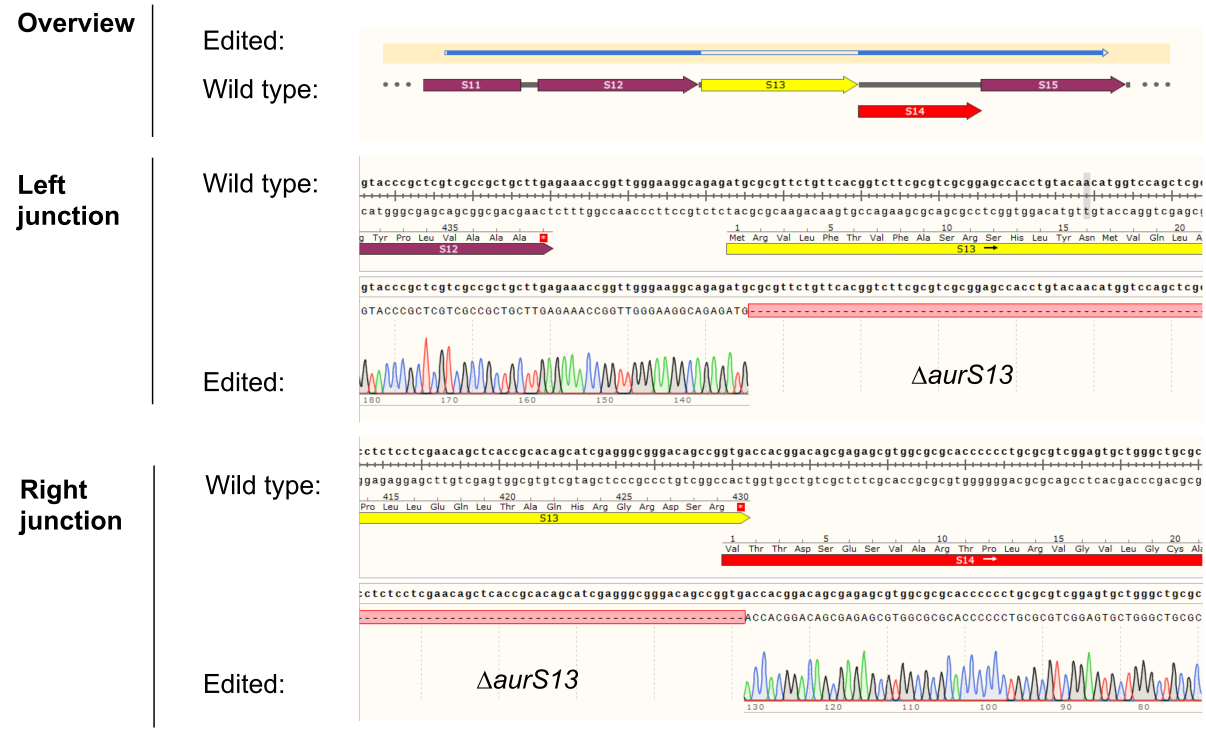

Figure S6. Alignment of sanger sequencing of *aurS13* deletion mutant (edited) against wild type genome.

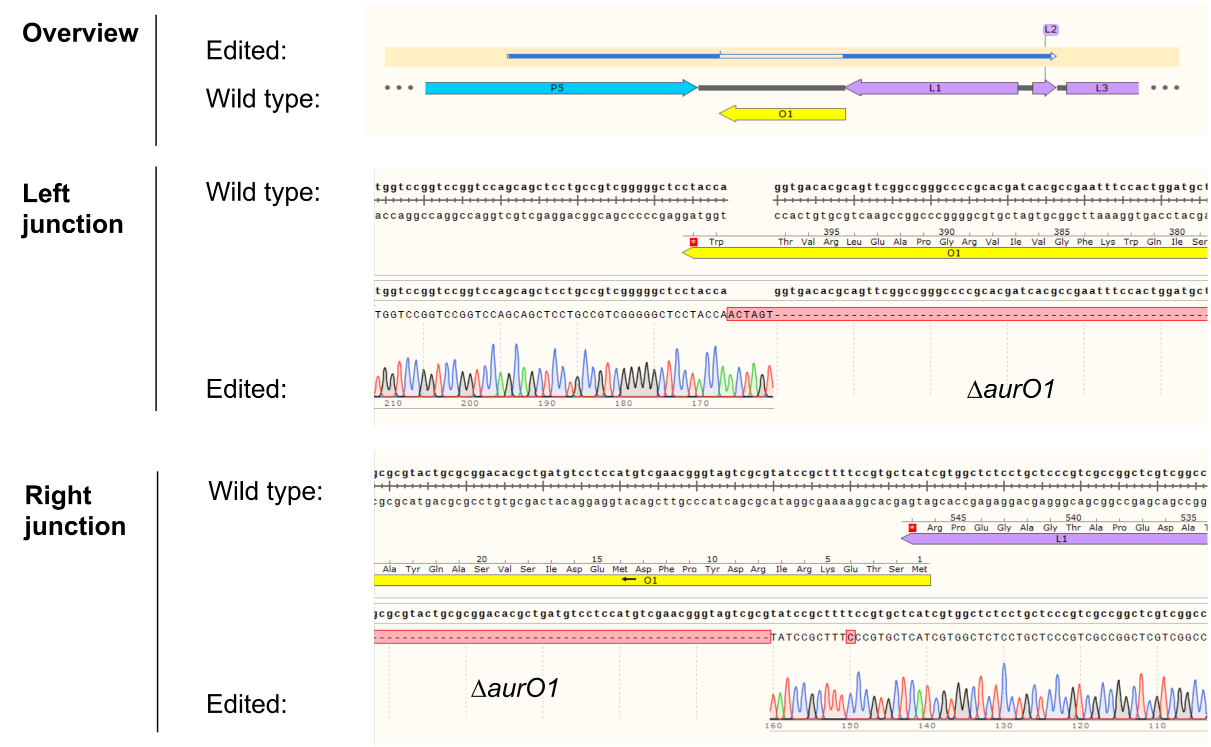

Figure S7. Alignment of sanger sequencing of *aurO1* deletion mutant (edited) against wild type genome. A *Spe*I site was inserted in place of the *aurO1* deletion. A single base mutation was also observed on the remaining segment of *aurO1*, however we predict minimal effects of this single base mutation on our experiments.

.

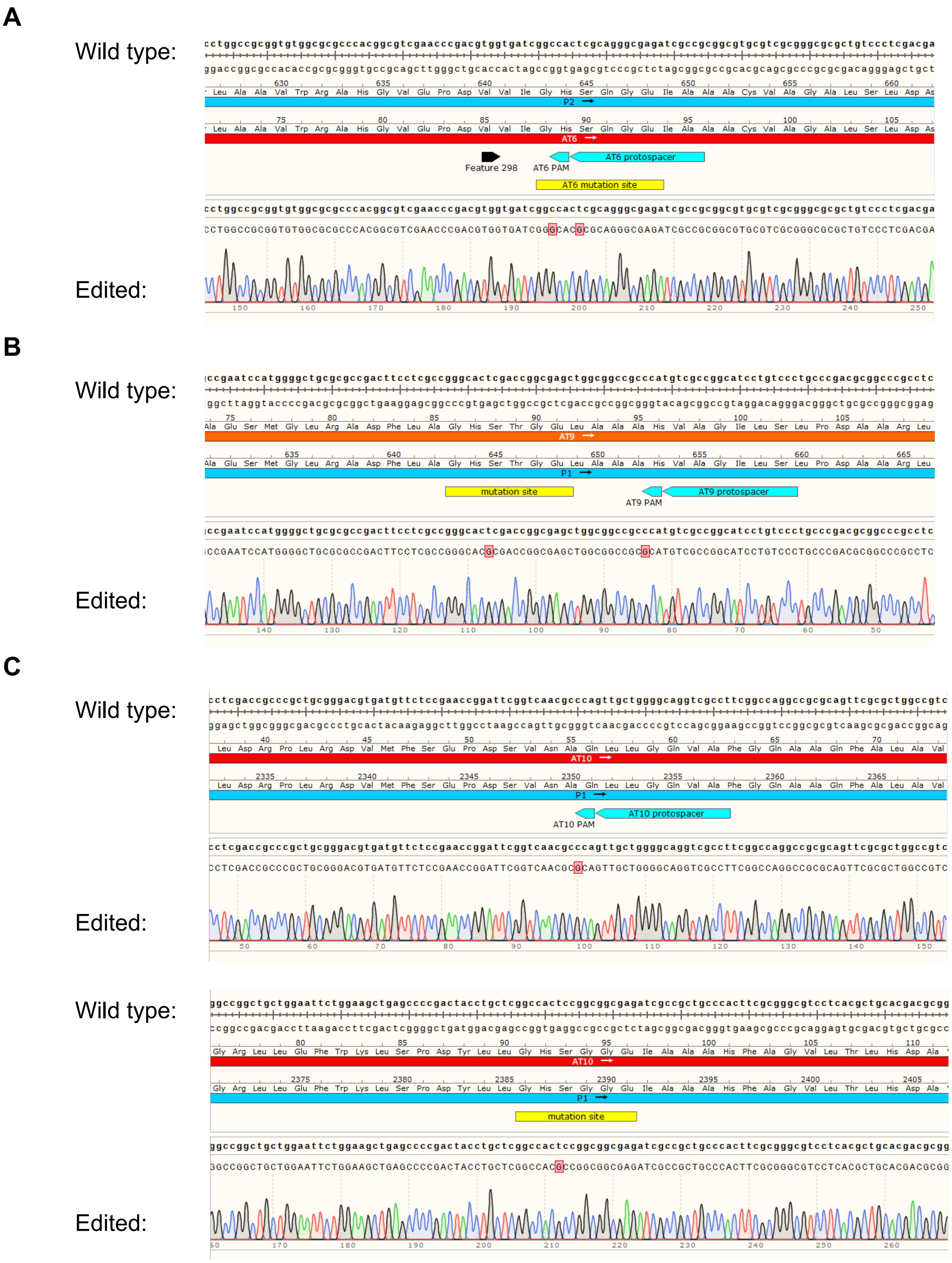

Figure S8. Alignment of sanger sequencing of AT mutants (edited) against wild type genome. Active site conserved serine to alanine mutants for (A) AT6 (B) AT9 and (C) AT10. PAM sites were also mutated to stop re-cutting by CRISPR-Cas.

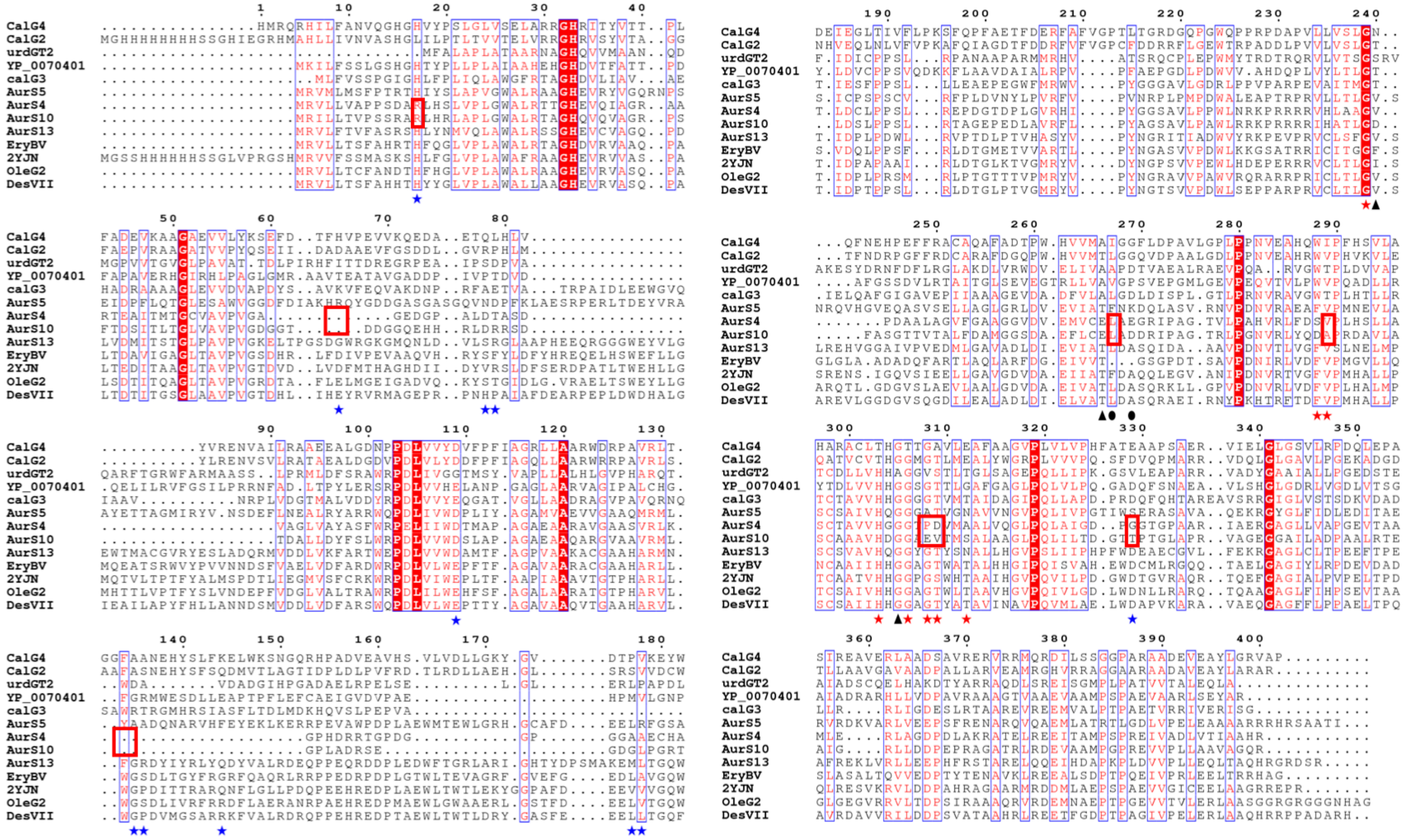

Figure S9 Alignment of glycosyltransferases. Putative residues, based on EryCIII (2YJN, Moncrieffe et al, 2012) involved in acceptor and donor nucleotide binding are highlighted. Residues marked with blue stars are involved in acceptor binding while residues marked with red stars are involved in both TDP and UDP binding. Black triangles and circles denote residues that are involved in only UDP or TDP binding. Distinct differences within the truncated glycosyltransferases for proposed residues participating in acceptor or sugar binding are boxed in red.

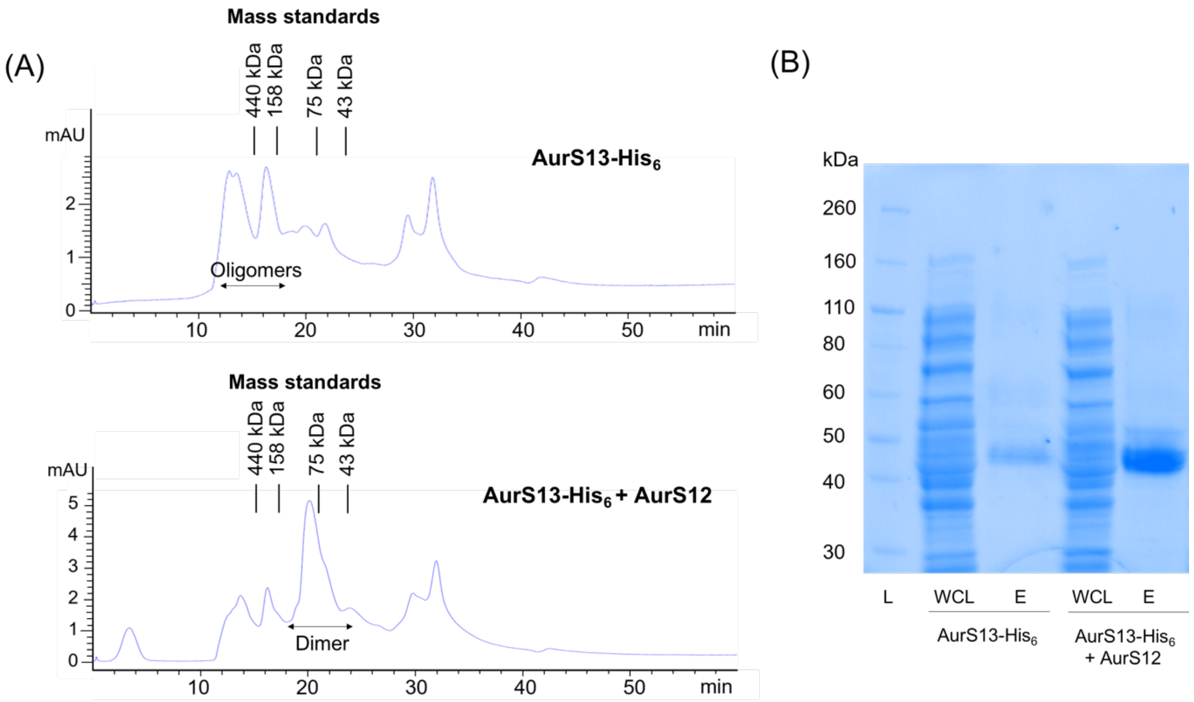

Figure S10 Purification and characterization of His-tagged AurS13 expressed alone or His-tagged AurS13 co-expressed with AurS12 (no His-tag). (A) Size exclusion chromatography (B) SDS-PAGE gel of Ni-NTA purification of His-tagged AurS13 expressed alone and His-tagged AurS13 co-expressed with AurS12 (no His-tag) (L: ladder, WCL: wash collection, E: Elution)

AurS12 and AurS13-His$_6$ were expressed and purified by Ni-NTA purification and anionic separation (FPLC, intact proteins), we observed a higher protein yield of AurS13 when co-expressed with AurS12. Size exclusion chromatography of AurS13 also showed a change from oligomers to dimer formation when the protein was co-expressed with AurS12. This is consistent with a previous study reporting conformation changes of DesVII in the presence of DesVIII (Borisova 2010).

Table S4. ^{1}H and ^{13}C NMR data of auroramycin analog **4** and analog **5**.

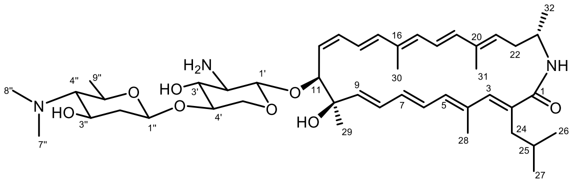

4

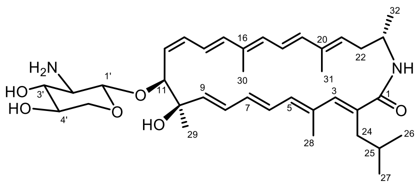

5

No.	**4** (CD_2Cl_2:CD_3OD = 5:1) δC	δH (multiplicity, *J* (Hz))	**5** (DMSO-d_6) δC	δH (multiplicity, *J* (Hz))
1	177.30, C		174.57,170.44,C	
2	135.67, C*		135.41, C*	
3		137.42, CH	136.19, CH	6.14 (1H, s)
4	133.56, C		131.97, C	
5	136.15, CH	5.78 (1H, m)	134.65, CH	5.70 (1H, d, 11.5)
6	128.63, CH	6.39 (1H, m)	127.44, CH*	6.45 (1H, m)
7	135.67, CH*	5.90 (1H, m)	134.87, CH	5.79 (1H, t, 12.8)
8	130.47, CH	6.21 (1H, m)	130.87, CH	6.12 (1H, m)
9	140.95, CH	5.51 (1H, m)	142.23, CH	5.47 (1H, d, 15.6)
10	76.33, C		75.51, C	
11	83.61, 88.89 CH	4.25 (1H, m)	82.29, CH	4.24 (1H, d, 8.3)
12	130.17, CH	5.49 (1H, m)	130.73, CH	5.40 (1H, m)
13	129.39, CH	6.16 (1H, m)	127.44, CH*	6.16 (1H, m)
14	125.57, CH	6.19 (1H, m)	125.56, CH	6.15 (1H, m)
15	138.11, CH	6.28 (1H, m)	135.41, CH*	6.20 (1H, m)
16	134.17, C		133.35, C	
17	132.02, CH	6.11 (1H, m)	127.54, CH	6.00 (1H, m)
18	124.45, CH	6.33 (1H, m)	123.14, CH	6.30 (1H, m)
19	137.64, CH	6.39 (1H, m)	137.24, CH	6.22 (1H, m)
20	136.64, C		137.69, C	
21	129.92, CH	5.58 (1H, m)	127.27, CH	5.68 (1H, m)
22	39.52, CH_2	1.12 (1H, m)<br>1.47 (1H, m)	35.84, CH_2	2.42 (2H, m)
23	46.88, 45.26 CH	3.97 (1H, m)	45.13, CH	3.93 (1H, m)
24	34.76, CH_2	2.23 (1H, t, 7.6)<br>2.49 (1H, m)	33.75, CH_2	2.17 (1H, m)
25	29.14, CH	1.61 (1H, m)	24.55, CH	1.57 (1H, m)
26	22.46, CH_3	0.83 (3H, m)^	22.29, CH_3	0.82 (3H, m)
27	22.54, CH_3	0.83 (3H, m)^	22.34, CH_3	0.84 (3H, d, 6.7)
28	16.38, CH_3	1.96 (3H, s)	16.09, CH_3	1.94 (3H, s)
29	20.96, CH_3	1.23 (3H, br s)^	23.62, CH_3	1.41 (3H, brs)
30	12.99, CH_3	1.71 (3H, s)	12.72, CH_3	1.71 (3H, s)
31	12.87, CH_3	1.73 (3H, s)	11.23 CH_3	0.80 (3H, m)
32	19.31, CH_3	1.23 (3H, br s)^	20.81, CH_3	1.17 (3H, d, 6.7)
1-NH				
1'	104.60, 106.80, CH	4.29 (1H, m)	105.37, CH	4.18 (1H, d, 7.6)
2'	57.67, CH	2.68 (1H, m)	57.74, CH	2.49 (1H, m)
3'	74.99, CH	3.31 (1H, m)	76.00, CH	3.02 (1H, m)
4'	79.69, CH	3.53 (1H, m)	69.59, CH	3.26 (1H, m)
5'	64.32, CH_2	3.22 (1H, t, 11.0)<br>3.89 (1H,dd, 5.4, 11.8)	66.00, CH_2	3.00 (1H, m)<br>3.66 (1H, dd, 5.2, 11.4)
1"	100.27, CH	4.48 (1H, dd, 2.0, 9.8)		
2"	39.79, CH_2	1.48 (1H, m)<br>2.20 (1H, ddd, 2.0, 4.8, 12.3)		
3"	71.05, CH	3,52 (1H, m)		
4"	72.04, CH	2.02 (1H, t, 9.8)		
5"	66.24, CH	3.62 (1H, ddd, 4.8, 9.8, 11.3)		
6"				
7"	41.55, CH_3*	2.40 (3H, s)^		
8"	41.55, CH_3*	2.40 (3H, s)^		
9"	19.96, CH_3	1.28 (3H, d, 6.2)		

***overlap ^{13}C signals; ^overlap ^{1}H signals; chemical shifts in ppm using CD_2Cl_2 (δ_H = 5.32 ppm, δ_C = 53.84 ppm) or DMSO-6 (δ_H = 2.50 ppm, δ_C = 39.52 ppm) as reference.**

Table S5. ^{1}H and ^{13}C NMR data of auroramycin analog **10**.

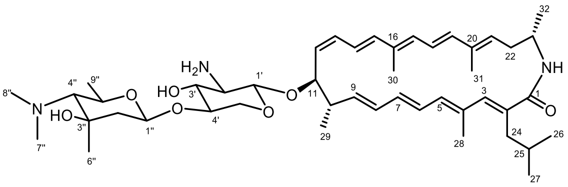

10

No.	**10** (CD_2Cl_2:CD_3OD = 5:1) δC	δH (multiplicity, *J* (Hz))
1	173.00, 177.15, C	
2	136.52, C	
3	136.89, CH	6.17 (1H, s)
4	132.40, C	
5	136.78, CH	5.80 (1H, m)
6	126.82, CH	6.33 (1H, m)^
7	136.52, CH	5.91 (1H, m)
8	131.69, CH	6.16 (1H, m)
9	140.04, CH	5.61 (1H, m)
10	28.41, CH	1.48 (1H, m)
11	70.66, CH	3.89 (1H, m)
12	130.06, CH	5.44 (1H, m)
13	129.18, CH	6.14 (1H, m)
14	124.57, CH	6.42 (1H, m)
15	137.30, CH	6.33 (1H, m)^
16	133.55, C	
17	131.44, CH	7.02 (1H, m)
18	115.65, CH	6.73 (1H, m)
19	137.46, CH	6.40 (1H, m)
20	137.40, C	
21	136.68, CH	6.20 (1H, m)
22	30.10, CH_2	1.24 (2H, m)^
23	46.86, CH*	3.96 (1H, m)^
24	36.94, CH_2	2.24 (1H, m)
		2.48 (1H, m)
25	29.29, CH	1.62 (1H, m)
26	22.53, CH_3	0.84 (3H, m)
27	22.73, CH_3	0.85 (3H, m)
28	16.33, CH_3	1.96 (3H, s)
29	23.10, CH_3	0.83 (3H, m)
30	12.90, CH_3	1.73 (3H, s)
31	12.97, CH_3	1.75 (3H, s)
32	23.32, CH_3	1.24 (3H, m)^
1-NH		
1'	104.76, CH	4.23 (1H, m)
2'	57.51, CH	2.71 (1H, m)
3'	74.84, CH	3.31 (1H, m)
4'	79.84, CH	3.52 (1H, m)
5'	64.34, CH_2	3.20 (1H, t, 11.3)
		3.87 (1H, m)
1"	99.74, CH	4.55 (1H, d, 9.9)
2"	45.83, CH_2	1.60 (1H, m)
		1.87 (1H, m)
3"	71.96, C	
4"	74.42, CH	2.24 (1H, m)
5"	69.97, CH	3.72 (1H, m)
6"	23.10, CH_3	1.24 (3H, s)^
7"	44.04, CH_3*	2.44 (3H, s)^
8"	44.04, CH_3*	2.44 (3H, s)^
9"	20.91, CH_3	1.30 (3H, d, *J* = 5.9 Hz)

***overlap ^{13}C signals; ^overlap ^{1}H signals; chemical shifts in ppm using CD_2Cl_2 (δ_H = 5.32 ppm, δ_C = 53.84 ppm) as reference.**

Figure S11. Analytical data for the structural assignment of auroramycin analog **3**.

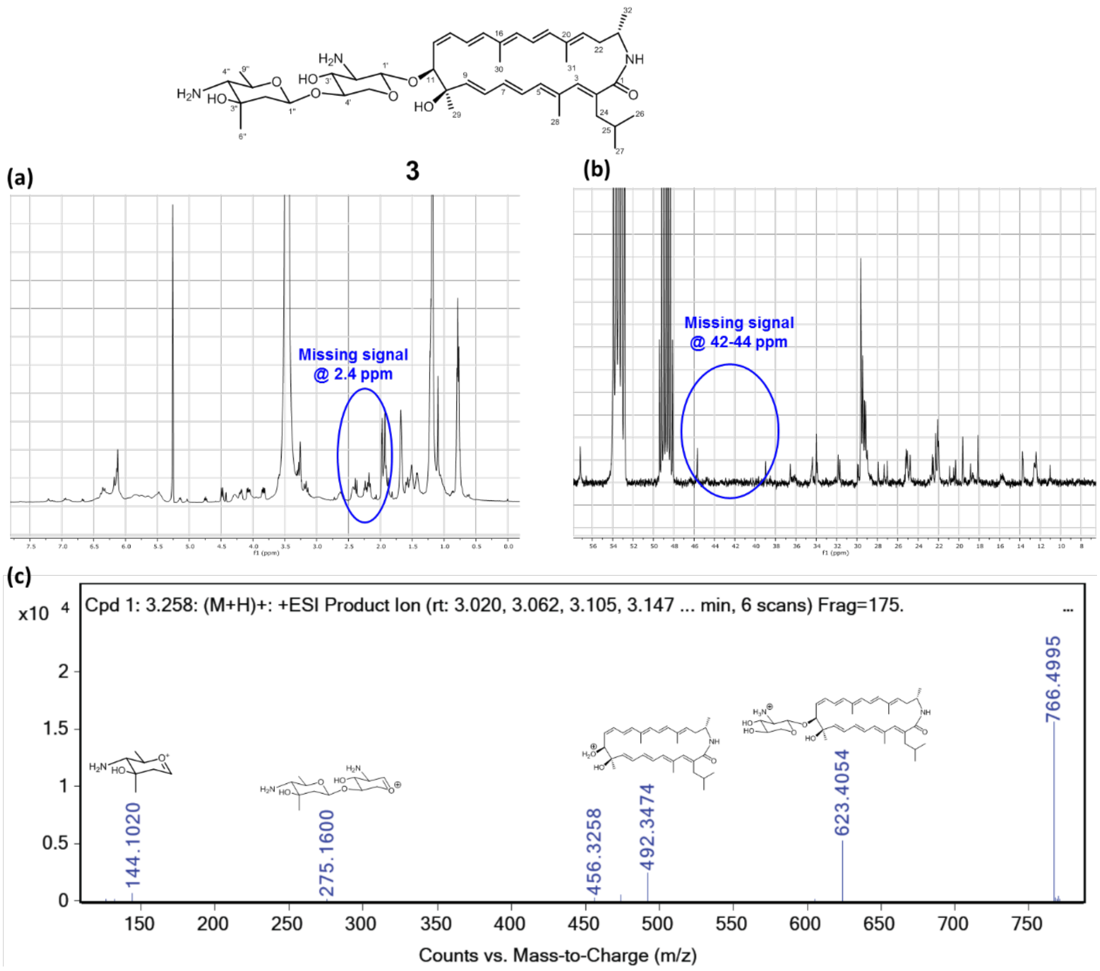

Structural assignment of auroramycin analog **3** was confirmed by combined inference of the NMR, HRMS and MS/MS data. Key features from the ^{1}H and ^{13}C that corroborate with the assigned structure are the disappearance of the methyl $\boldsymbol{\delta_H}$ and $\boldsymbol{\delta_c}$ signals at around 2.4 ppm and 42-44 ppm respectively (Figure S11a and S11b). This is further supported by the MS/MS data in which the MS2 signal 144.1020 can be assigned to the *N*,*N*-demethylated 3, 5-*epi*-lemonose unit (Figure S11c).

Figure S12. Analytical data for the structural assignment of auroramycin analog **6** and minor hydroxylated product.

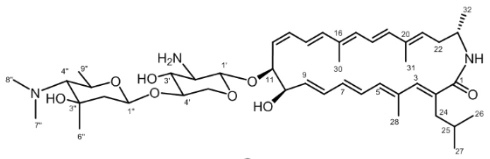

6

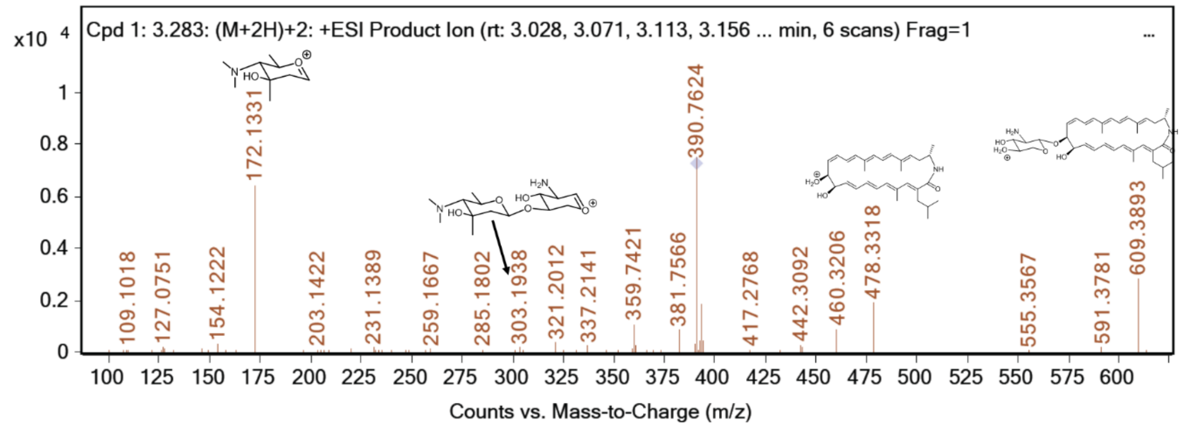

Structural assignment of auroramycin analog **6** was confirmed by inference of the HRMS and MS/MS data. Key features of the MS2 spectra has been assigned. MS2 signal 478.3318 can be assigned to the macrolactam fragment which suggests a methyl group is missing in analog **6** compared to auroramycin.

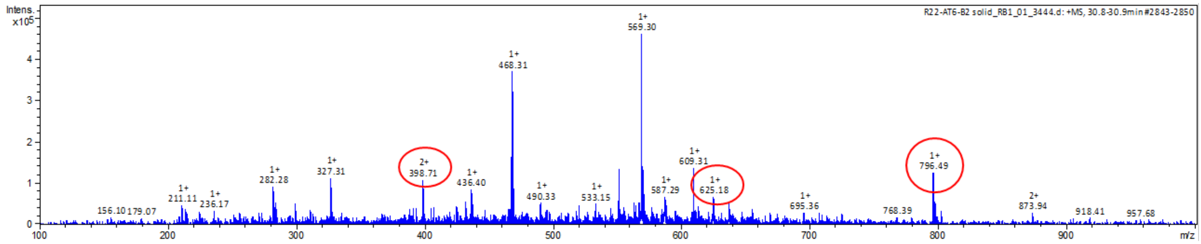

The minor hydroxylated product had a m/z of 796.49 which is 16 Da more than the mass of the expected analog **6**.

Figure S13. Analytical data for the structural assignment of auroramycin analog **7** and minor hydroxylated product.

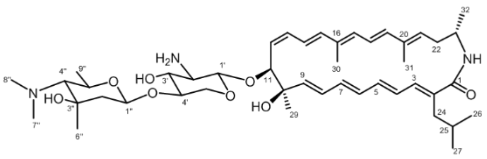

7

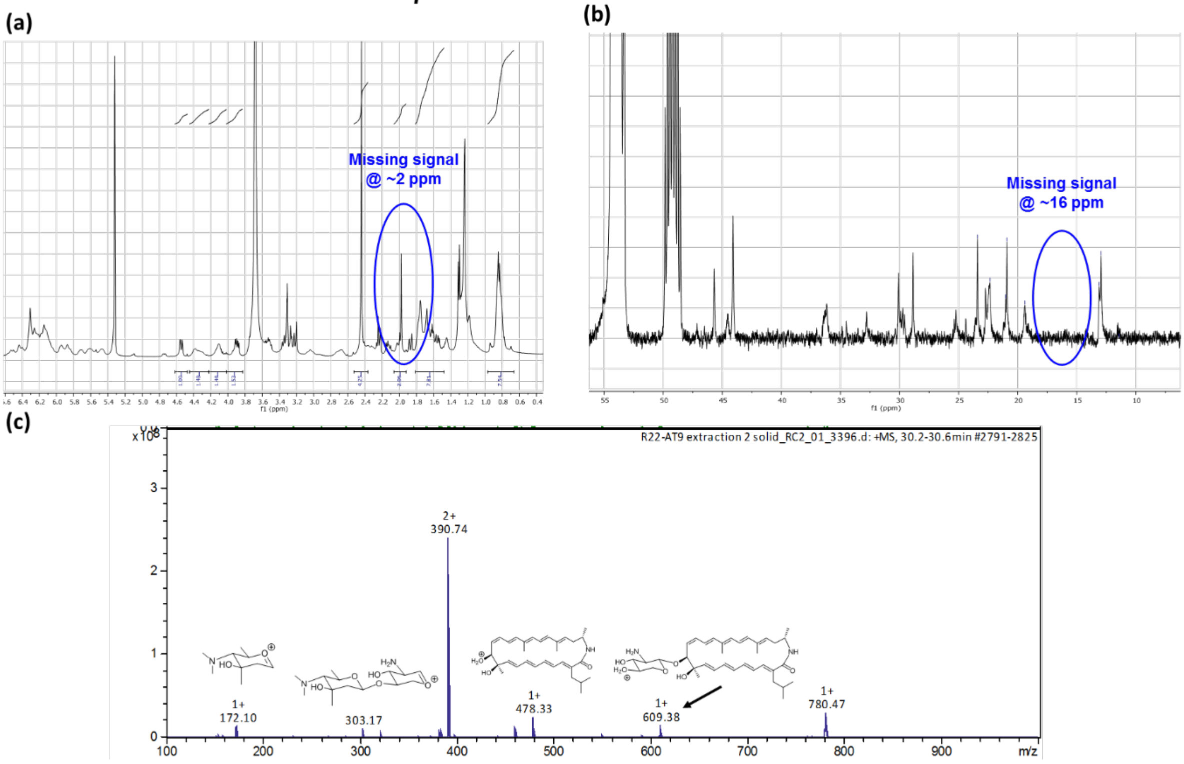

Structural assignment of auroramycin analog **7** was confirmed by combined inference of the NMR, HRMS and MS/MS data. Key features from the ^{1}H and ^{13}C that corroborate with the assigned structure are the disappearance of the methyl δ_H and δ_c signals at around 2 ppm and 16 ppm respectively (Figure S13a and S13b). This is further supported by the MS/MS data in which the MS2 signal 478.33 can be assigned to the macrolactam fragment which suggests a methyl group is missing in analog **7** compared to auroramycin (Figure S13c).

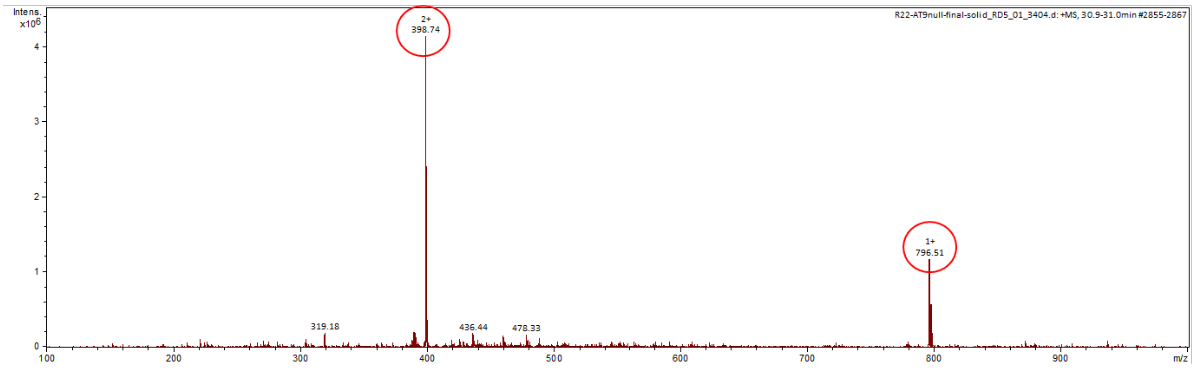

The minor hydroxylated product had a m/z of 796.51 which is 16 Da more than the mass of the expected analog **7**.

Figure S14. Representative microdilution assay plates for compound **1**, **3**-**7**, **10** with (A) *Staphylococcus aureus* N216, (B) *S. aureus* Z172, (C) *Enterococcus faecalis* ATCC 51299, (D) *Pseudomonas aeruginosa* ATCC 700603 and (E) *E. coli* ATCC 25922. *The compound **3** tested in this MIC assay contains analog **5** in a 7:1 ratio. **Compound **7** is in a 5:1 ratio with a hydroxylated component.

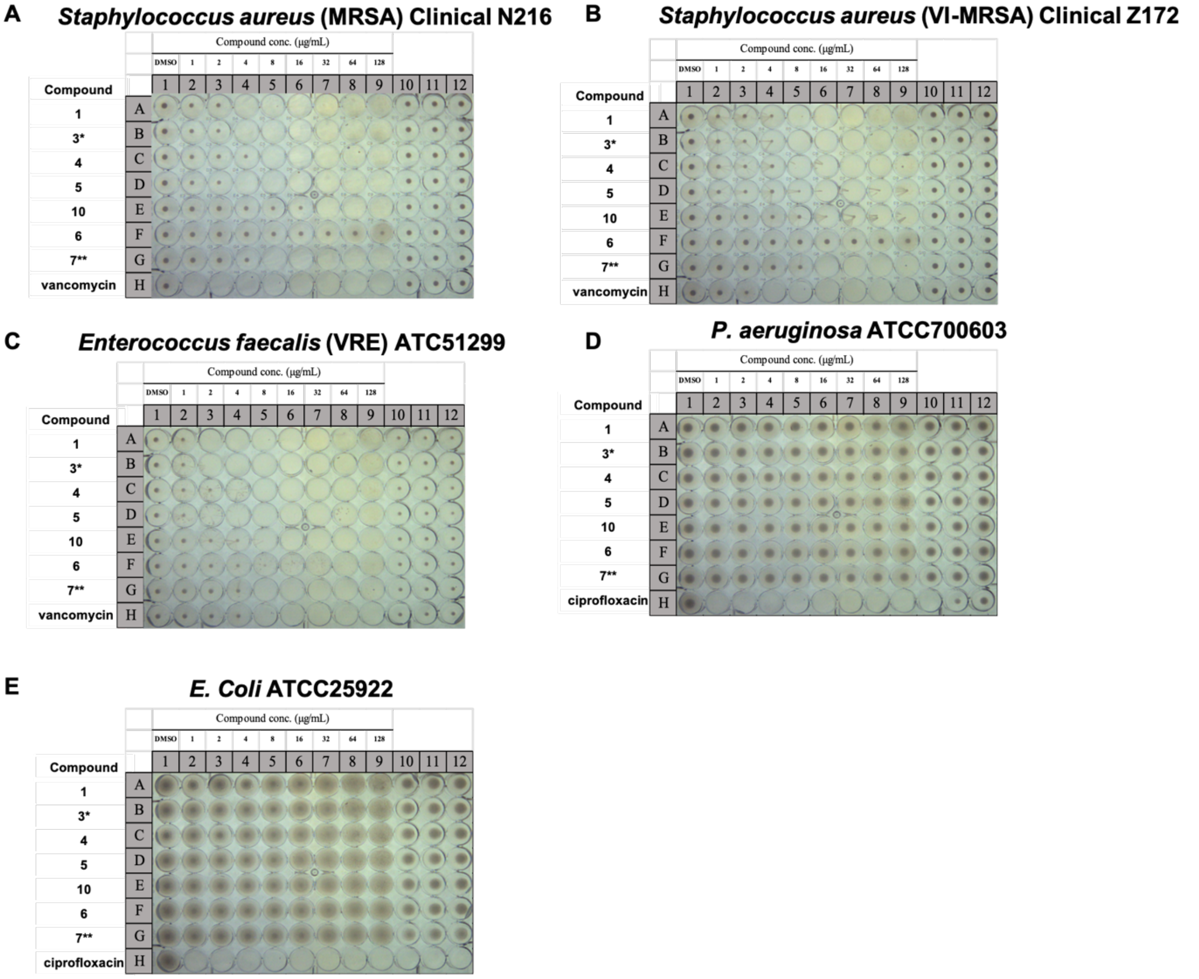

References:

Moncrieffe MC, Fernandez MJ, Spiteller D, Matsumura H, Gay NJ, Luisi BF, Leadlay PF. Structure of the glycosyltransferase EryCIII in complex with its activating P450 homologue EryCII. Journal of molecular biology. 2012 Jan 6;415(1):92-101.

Borisova SA, Liu HW. Characterization of glycosyltransferase DesVII and its auxiliary partner protein DesVIII in the methymycin/pikromycin biosynthetic pathway. Biochemistry. 2010 Aug 24;49(37):8071-84.
